# Cost-effectiveness of ten commonly used antipsychotics in first-episode schizophrenia in the UK: economic evaluation based on a *de novo* discrete event simulation model

**DOI:** 10.1192/bjp.2024.251

**Published:** 2025-08

**Authors:** Junwen Zhou, Aurelie Millier, Samuel Aballea, Clement Francois, Huajin Jin, Ryan Williams, Belinda Lennox, Apostolos Tsiachristas, Mondher Toumi

**Affiliations:** Public Health Department, Aix Marseille University, Marseille, France; Health Economic Research Centre, Nuffield Department of Population Health, University of Oxford, Oxford, UK; Clever-Access, Paris, France; King's Health Economics, Institute of Psychiatry, Psychology & Neuroscience at King's College London, London, UK; Institute for Global Health and Development, Peking University, Beijing, China; Division of Psychiatry, University College London, London, UK; Division of Psychiatry, Imperial College London, London, UK; Nuffield Department of Primary Care Health Sciences, University of Oxford, Oxford, UK; Department of Psychiatry, University of Oxford, Oxford, UK

**Keywords:** Schizophrenia, antipsychotics, economic evaluation, cost–utility analysis, discrete event simulation

## Abstract

**Background:**

Previous economic evidence about interventions for schizophrenia is outdated, non-transparent and/or limited to a specific clinical context.

**Aims:**

We developed a *de novo* discrete event simulation (DES) model for estimating the cost-effectiveness of interventions in schizophrenia in the UK.

**Method:**

The DES model was developed based on the structure of previous models, populated with demographic, clinical and cost data from the UK, and antipsychotics' effects from recent network meta-analyses. We simulated treatment pathways for patients with first-episode schizophrenia including events such as relapse, remission, treatment discontinuation, cardiovascular disease and death and estimated costs (2020£) taking the National Health Service perspective and quality-adjusted life years (QALYs) over ten years. Using the model, we ranked ten first-line antipsychotics based on their QALYs and cost-effectiveness.

**Results:**

Amisulpride was associated with the highest QALYs, followed by risperidone long-acting injection (LAI), aripiprazole-LAI (6.121, 6.084, 6.070, respectively) and others (5.947–6.058). The most cost-effective antipsychotics were amisulpride, olanzapine and risperidone-LAI, with total probability of rankings of 1, ≤2, ≤3, that is, 95%, 89%, 80%, respectively; meanwhile, the least cost-effective were cariprazine, lurasidone and quetiapine, with total probability of rankings of 10, ≥9, ≥8, that is, 96%, 92%, 81%, respectively. Results were robust across sensitivity analyses and influenced primarily by relapse relevant parameters.

**Conclusions:**

Our findings suggest amisulpride (or risperidone-LAI where oral treatment is inappropriate) as the best overall first-line option based on QALYs and cost-effectiveness. Our ranking may be used to guide decision-making between antipsychotics. Our model is open source and could be applied to the other settings.

Despite its relatively low prevalence, schizophrenia is in the top ten chronic conditions with the highest health and economic impact worldwide.^1^ Patients who achieve remission after their first episode still have the potential for multiple further episodes, and are usually treated with long-term antipsychotics to prevent relapse.^2^ For many, symptoms will impair activities of daily living, adversely affect health and require lengthy hospital admissions.^3^ Considering the vast majority of patients with schizophrenia experience their first psychotic episode by the age of 25,^3^ the negative impact of schizophrenia on health and healthcare budgets accumulates over many decades during each patient's lifetime. The choice of antipsychotics is based on the trade-off between efficacy and adverse effects. In the UK, clinicians and patients are theoretically able to choose between more than 20 antipsychotics currently available for the treatment of schizophrenia.^4^ Economic evidence has the potential to inform clinician and patient choice by taking multi-dimensional treatment profiles into account (e.g. efficacy, side-effects and drug cost). Cost-effectiveness evidence provides the expected net utility of an antipsychotic over a period, which is the utility gained from the drug efficacy after deducting the dis-utilities from the anticipated adverse events. From a funder's perspective, choice of antipsychotic agent based on such evidence could improve efficiency and affordability in treating schizophrenia. From the perspective of clinicians and patients, cost-effectiveness evidence simplifies the decision problem by synthesising treatment effects on multiple outcomes into the effect on one single outcome (e.g. quality-adjusted life year, QALY).

However, current economic evidence regarding antipsychotics is limited and weak. Most cost-effectiveness studies were designed to compare two antipsychotics at a time^5,6^ and do not capture patient heterogeneity as they modelled outcomes for a cohort of homogeneous patients (i.e. cohort-level models). To date, there have been four core patient-level models for the economic evaluation of antipsychotics in schizophrenia^7–10^ (and one core whole disease pathway model, but not specific for such evaluation^11^). However, all of these had substantial methodological limitations. For example, Heeg et al^9^ did not model explicitly long-term comorbidities and the remaining models^7,8,10^ did not consider the heterogeneity of relapse risks according to individual characteristics.^12^ In addition, they were developed more than ten years ago and are not able to reflect the changes to current clinical practice or incorporate recent evidence. Moreover, these existing patient-level models are subject to criticism as they are not open source, with this lack of transparency affecting the ability to critically appraise such models and assess validity.^13^ In this study, we developed a *de novo* core discrete event simulation (DES) model for the economic evaluation of antipsychotics in schizophrenia. The model was aimed to include the important features considered in the previous models, be flexible for further adaptation in model inputs and/or structures to other settings and be open source to increase transparency and allow further use by others.^14^ The developed model was demonstrated in an economic evaluation, comparing first-line antipsychotics in first-episode schizophrenia in the UK.

## Method

### Overview

DES models are used in economic evaluations to simulate the disease trajectory and series of health events of individual patients. At the occurrence of each event, the model advances the modelling time from the previous event to the current event, and simulates the individual's status, the next event to occur and the time to the next event based on the current event and the individual's status before the current event.^13,15^ Compared to other models for economic evaluation (e.g. Markov model), DES model accounts for patient heterogeneity and performs better in terms of accuracy, reliability and speed.^16^ Here, we describe a simulated cohort study of evaluating first-line antipsychotics for patients with first-episode schizophrenia in the UK using our model (the base-case for the model parameters and mathematical inter-relations). It should be noted that further adaptation and application would also be possible by changing the model inputs and/or structures to reflect the pattern in the other contexts.

### Analytical framework

The target population was treatment-naïve patients with first-episode schizophrenia eligible for antipsychotics. We simulated a cohort of 1000 participants with first-episode schizophrenia, based on patient profiles from a cross-sectional study in the UK by Smith et al^17^ (Supplementary Section 1 available at https://doi.org/10.1192/bjp.2024.251). Smith et al was chosen for two reasons: (a) it is the only available UK study reporting the model-needed cardio-metabolic profiles of patients with the closest diagnosis (first-episode psychosis) to first-episode schizophrenia^17^; (b) its summary of age, gender and total cholesterol are similar to those reported in a trial recruiting European patients with first-episode schizophrenia. Although this trial also reported the other metabolic profiles, only the categorised profiles were reported (e.g. the proportion of having triglycerides >150 mg/dl, but not the mean and s.d. of the triglycerides).^18^

The target comparison was between antipsychotics as the first-line treatment for relapse prevention. We compared ten atypical antipsychotics (seven oral antipsychotics: amisulpride, aripiprazole, cariprazine, lurasidone, olanzapine, quetiapine, risperidone; three long-acting injection (LAI) antipsychotics: aripiprazole, paliperidone, risperidone). These were selected following discussions with clinicians with expertise in schizophrenia and represent the most commonly prescribed antipsychotics in the UK (except cariprazine, which only became available in 2018) (Supplementary Table 1).^19^ Olanzapine-LAI was not included as it is much less frequently used than the other LAIs because of side-effects, the relatively high risk of ‘post-injection syndrome’ and increased monitoring requirements.^20^ Collectively these factors mean that olanzapine-LAI is not currently used in an equivalent way to other LAI agents in routine clinical practice – in many UK services it is available only as a ‘non-formulary’ medication for specialist use – reflected in extremely low relative prescription rates (94% lower than the next most commonly prescribed LAI agent in 2022). LAI agents were included alongside oral agents in the ‘first-line’ group. This is because although patients with schizophrenia would typically be offered an oral agent first, there are a variety of situations where a LAI could be used ‘first-line’ (patient preference, or for those unable or unwilling to adhere to oral treatment) – and this strategy is increasingly recommended.^21^

The target outcomes were QALYs and costs over a time horizon of ten years, which was long enough to capture differences in efficacy, side-effects and the associated costs between compared antipsychotics, but not too long to risk losing prediction precision. QALYs and costs were discounted at an annual rate of 3.5%.^22^

### Model structure

The structures of the previous models for economic evaluation of antipsychotics in schizophrenia^5^ were reviewed and are summarised in Supplementary Section 2. The DES model was built to simulate the commonly considered pathways to extrapolate the difference between antipsychotics in their treatment attributes to their long-term difference in costs and QALYs, including (a) disease progression, (b) treatment sequence, (c) treatment side-effects and (d) survival progression.

The following patient characteristics were included: disease status (stable or relapse), treatment status, age, gender, metabolic profiles (body mass index (BMI), total cholesterol, high-density lipoprotein cholesterol (HDL-C), triglyceride, fasting glucose and systolic blood pressure), behaviour factors (levels of adherence to medication, smoking status, alcohol consumption) and history of treatment and disease conditions such as prior coronary heart disease (CHD), stroke and diabetes. The following events were included to advance the modelling time: relapse, remission, treatment discontinuation, tardive dyskinesia (a neurological disorder specifically associated with antipsychotic use), first CHD, first stroke, diabetes and death. Relapse was defined as a new ‘psychotic episode’ – a recurrence of or deterioration in psychotic symptoms relative to baseline by clinical judgement, which is used in most randomised controlled trials (RCTs) for assessing efficacy of antipsychotics in relapse prevention.^23^ Remission was defined as the end of a relapse episode.^3^

**Fig. 1:**
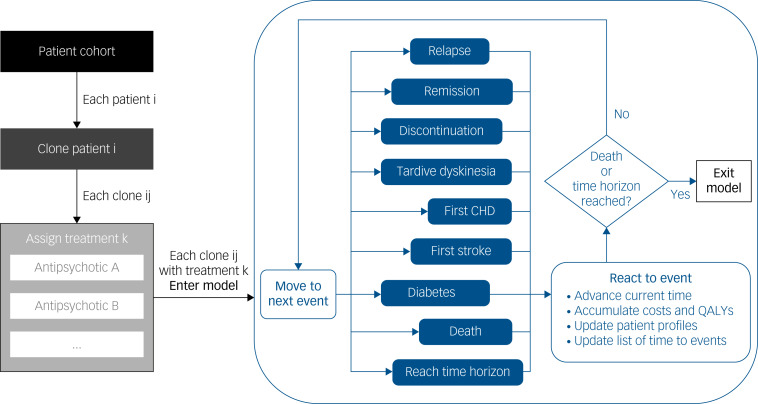
Overview of model structure. ‘Relapse’, ‘remission’ and ‘discontinuation’ can occur multiple times, whereas the other events can only occur once. CHD, coronary heart disease; QALYs, quality-adjusted life years.

Figure 1 depicts the model structure. Patients entered the model in a stable status and were prescribed an antipsychotic for relapse prevention – one of the ten included ‘first-line’ antipsychotics. Patients might then discontinue treatment, which increased their risk of relapse, and then relapse. If they relapsed, they might remain on the current treatment, or switch to the next line of treatment. For those who switched, the ‘second-line’ treatments comprised the remaining nine antipsychotics not yet tried, with equal weight. Again, patients might relapse (+/– discontinue) and switch treatment. Following current clinical practice, patients in the model were considered treatment-resistant and were prescribed clozapine third and last line after two consecutive failures of other antipsychotics.^3^ Treatment switch owing to side-effects was not explicitly modelled as limited data is available to model such an event, and patients are usually recommended to remain on treatment if the treatment is still effective for them. In this model, we focused on patients who can tolerate their assigned treatment at the treatment start, and modelled treatment switch owing to side-effects over the long term implicitly through all-cause treatment discontinuation, including also those owing to patient preference, which led to increased risk of relapse potentially resulting in a treatment switch.

Treatment side-effects were categorised into metabolic, short-term and long-term side-effects. Metabolic side-effects (changes in BMI, total cholesterol, HDL-C, triglyceride, fasting glucose and systolic blood pressure) were included for modelling the long-term risk of diabetes and cardiovascular disease, that is, it was assumed that they did not require immediate treatment nor have an immediate impact on quality of life, but that they increased the risk of diabetes and cardiovascular disease. Low-density lipoprotein cholesterol was not included because it is usually not used alongside total cholesterol in predicting cardio-metabolic outcomes.

Both short- and long-term side-effects required treatment and/or caused a decrement in quality of life. Short-term side-effects were unlikely to occur beyond 3 months after receiving treatment, including agranulocytosis (only for clozapine), acute extrapyramidal side-effects (EPS), sedation, sexual dysfunction and weight gain. Long-term side-effects were those that continued to occur beyond 3 months after receiving treatment, and including tardive dyskinesia and first occurrence of CHD, stroke and diabetes.

Throughout the simulation, patients might experience some or all of the following events: first occurrence of CHD, stroke and diabetes and death. Metabolic side-effects were assumed to occur and disappear once the patients started and discontinued antipsychotics, respectively, which would affect the time to CHD, stroke and diabetes. Short-term side-effects were assumed to occur in a certain proportion of patients based on predefined distributions after initiation of antipsychotics.

### Model inputs

Model input parameters included the following (a) treatment attributes, including efficacy, safety, tolerability and cost; (b) epidemiological parameters (e.g. the coefficients of the model for time to CHD); (c) health-related quality of life; and (d) costs (other than drug costs). They were pre-filled with the UK data to facilitate model development, with the flexibility to be replaced for the applications in the other contexts.

Table 1 summarises the attributes of the compared antipsychotics and clozapine. The efficacy, safety and tolerability profiles were primarily informed from the 2022 network meta-analyses (NMAs) by Schneider-Thoma et al,^23^ metabolic impacts were from the 2020 NMA by Pillinger et al^24^ and costs were from the British National Formulary (BNF) 79.^4^

**Table 1:** Attributes of antipsychotics

Treatment attribute	AMI	ARI (LAI)	CAR	LUR	OLA	PAL-LAI	QUE	RIS (LAI)	CLO
Relative risk of relapse versus placebo	0.19	0.32	0.65	0.63	0.20	0.31	0.47	0.29 (0.25)	0.06
Annual probability of discontinuation	0.25	0.46 (0.36)	0.61	0.51	0.27	0.43	0.42	0.31 (0.34)	0.33
Mean change in metabolic profiles									
Body mass index (kg/m^2^)	1.66	−0.22	0.83	0.24	1.07	0.56	0.70	0.56	1.02
Total cholesterol (mg/dL)	8.11	2.32	−3.47	−1.16	15.00	2.32	12.00	2.32	22.00
HDL cholesterol (mg/dL)	−3.86	1.54	0.77	0.77	−0.39	0.39	0.39	0.39	−4.48
Triglycerides (mg/dL)	7.96	1.77	0.88	0.00	41.00	3.54	28.00	3.54	87.00
Fasting glucose (mg/dL)	−8.29	2.34	4.68	−5.23	3.60	1.44	1.62	1.44	19.00
Systolic blood pressure (mmHg)	1.17	0.84	4.17	−1.59	1.28	2.60	2.60	2.60	3.90
Probability of short-term side-effects									
Extrapyramidal symptoms	0.28	0.41 (0.45)	0.36	0.19	0.04	0.35	0.21	0.26 (0.27)	0.06
Sedation	0.03	0.01 (0.04)	0.01	0.02	0.03	0.04	0.06	0.02 (0.01)	0.15
Sexual dysfunction	0.03	0.27	0.01	0.00	0.40	0.57	0.15	0.45	0.53
Weight gain	0.27	0.13	0.21	0.19	0.36	0.18	0.40	0.18	0.26
Annual probability of tardive dyskinesia	0.02	0.02	0.05	0.05	0.03	0.02	0.02	0.02	0.04
Annual costs (£)	86	24 (2645)	1048	1183	23	3772	93	160 (1375)	868

AMI, amisulpride; ARI, aripiprazole; CAR, cariprazine; CLO, clozapine; HDL, high-density lipoprotein; LAI, long-acting injection; LUR, lurasidone; OLA, olanzapine; PAL, paliperidone; QUE, quetiapine; RIS, risperidone.

Treatment is an oral one if its name is not followed by ‘LAI’.

The treatment attributes were used together with the epidemiological parameters to simulate the long-term consequences. Time to relapse was assumed to follow an exponential distribution with annual probability of the event under no treatment informed from the NMAs by Schneider-Thoma et al^23^ and affected by the use of antipsychotics and the level of patient adherence to treatment with antipsychotics (patient using LAI was assumed to be fully adhering to antipsychotics treatments) over time. Time to remission was 6 months following the National Institute for Health and Care Excellence (NICE) model.^3^ Time to discontinuation and time to tardive dyskinesia were both assumed to follow exponential distribution based on treatment-specific annual probability. Time to first CHD, stroke and diabetes were simulated based on the Framingham scores of simulated patients, which were calculated using their characteristics.^25–27^ Time to death was simulated based on the national life table and the standard mortality ratio by schizophrenia^28^ in the UK. Published evidence informed additional risk of death at the occurrence of agranulocytosis,^29^ CHD^30^ and stroke,^31^ respectively. Patients were assumed to switch to the next line when they relapsed with an assumed probability of 0.5.

Utility weights for disease status were derived from Lenert et al,^32^ which is a commonly used source for such evidence in schizophrenia.^33^ Utility decrements owing to side-effects were primarily derived from studies that included the UK schizophrenia population. Costs were analysed from the UK National Health Service (NHS) perspective, including cost of primarily schizophrenia-related healthcare service, management of side-effects and death. The costs were either derived from the total costs or the frequency of resource use and their unit costs from published UK data and studies, converted to 2020 UK£.^34^ Supplementary Table 2 shows all the model inputs and their sources.

### Simulation algorithms

We followed best practice to perform the DES simulation.^15^ For each individual, a list of times to each possible event was generated based on the patient characteristics and treatment scenario. The simulation then progressed through a loop: (a) the earliest event was selected to occur; (b) the patient characteristics were updated after the event; (c) the list of times to each event was also updated. The loop continued until the model reached the time horizon or until the patient died. Supplementary Section 3 illustrates the simulation process of time to one single event. To ensure accuracy and stability, we ran 100 simulations for each patient, resulting in a total simulation of 100 000 individuals.

### Cost-effectiveness and uncertainty analyses

We performed the simulation for each of the 90 treatment sequences (ten first-line antipsychotics with each followed by nine second-line antipsychotics). The results for each first-line antipsychotic were generated from the mean across the results of the sequences with this first-line antipsychotic. We compared costs and QALYs for all pairs of comparison, using the one leading to lower QALYs as reference, and generated an incremental cost-effectiveness ratio (incremental costs / incremental QALYs). We ranked antipsychotics based on the monetary benefit calculated using total QALYs, willingness to pay (WTP) thresholds and total costs (monetary benefit = QALYs × WTP – costs). Ranking was present across WTPs from £0/QALY to £100 000/QALY. Probabilistic sensitivity analyses with 1000 simulations were performed to show the uncertainty in the ranking. Deterministic sensitivity analyses were performed for each pair of comparisons to identify the key drivers among all the model parameters for net monetary benefit at WTP of £20 000/QALY and the incremental QALYs. Scenario analyses were performed to explore the impact on ranking by different timeframes, discount rates and comparison scenarios (comparing antipsychotics as second-line treatment in non-first-episode schizophrenia (Supplementary Section 1)).

Supplementary Section 4 illustrates the use of the DES model, and an RShiny webpage version 1.9.1 for Windows (https://github.com/rstudio/shiny) was developed to facilitate the use (available at https://livedataoxford.shinyapps.io/shiny_des_schizophrenia/). All the analyses were performed in R version 4.2.2 for Windows (R Foundation for Statistical Computing, Vienna, Austria; https://www.R-project.org/).

## Results

Among the ten compared first-line antipsychotics, amisulpride resulted in the highest QALYs (discounted: 6.121), followed by risperidone-LAI (6.084), aripiprazole-LAI (6.070) and others (5.947–6.058). Amisulpride led to the largest years in stable state following first-episode schizophrenia and total QALYs excluding the loss owing to side-effects (undiscounted: 8.132 and 7.519, respectively), whereas cariprazine led to the smallest values in both (7.669 and 5.947, respectively). Total loss of QALYs owing to CHD, stroke or diabetes ranged from −0.310 with olanzapine to −0.247 with amisulpride; total loss of QALYs owing to the other side-effects ranged from −0.071 with cariprazine to −0.056 with lurasidone (Table 2)

**Table 2:** Base-case estimated outcomes over ten years under compared first-line antipsychotics

Outcome	AMI	RIS-LAI	ARI-LAI	OLA	PAL-LAI	RIS	ARI	LUR	QUE	CAR
Ranking from highest to lowest QALYs, discounted	1	2	3	4	5	6	7	8	9	10
Total QALYs, discounted	6.121	6.084	6.070	6.058	6.051	6.047	6.026	5.997	5.990	5.947
Total QALYs, undiscounted	7.213	7.169	7.155	7.137	7.133	7.129	7.107	7.079	7.067	7.021
QALYs related to schizophrenia state	7.519	7.506	7.485	7.511	7.473	7.470	7.442	7.400	7.421	7.395
QALY loss owing to CHD, stroke or diabetes	−0.247	−0.277	−0.275	−0.310	−0.278	−0.280	−0.277	−0.250	−0.291	−0.303
QALY loss owing to other side-effects^a^	−0.059	−0.060	−0.056	−0.063	−0.061	−0.062	−0.058	−0.071	−0.062	−0.071
Life years	9.905	9.907	9.907	9.905	9.905	9.905	9.903	9.905	9.904	9.903
Years in stable state of schizophrenia	8.132	8.077	8.001	8.097	7.956	7.948	7.843	7.685	7.766	7.669
Ranking from lowest to highest costs, discounted	1	3	5	2	7	4	6	9	8	10
Total costs (£), discounted	147 197	153 730	160 694	149 092	164 987	157 399	162 801	173 709	167 474	174 442
Total costs (£), undiscounted	174 163	181 548	189 109	176 304	193 762	185 292	191 074	202 103	195 812	202 844
Costs owing to antipsychotic treatments	4866	8885	12 230	4753	14 424	5590	5557	7826	5971	7525
Costs owing to schizophrenia-related healthcare	167 599	170 830	175 031	169 602	177 475	177 855	183 662	192 581	187 931	193 349
Costs owing to CHD, stroke or diabetes	1407	1534	1518	1682	1536	1542	1524	1407	1596	1644
Costs owing to other side-effects^a^	220	229	259	196	256	234	259	218	242	254
Costs owing to death	71	70	71	71	71	71	72	71	72	72
Ranking from most to least cost-effective at WTP £20 000/QALY	1	3	5	2	7	4	6	8	9	10

AMI, amisulpride; ARI, aripiprazole; CAR, cariprazine; CHD, coronary heart disease; LAI, long-acting injection; LUR, lurasidone; OLA, olanzapine; PAL, paliperidone; QALY, quality-adjusted life year; QUE, quetiapine; RIS, risperidone; WTP, willingness to pay.

a. Other side-effects include extrapyramidal symptom, weight gain, sedation, sexual dysfunction and tardive dyskinesia.

Treatment is an oral one if its name is not followed by ‘LAI’.

.

**Fig. 2:**
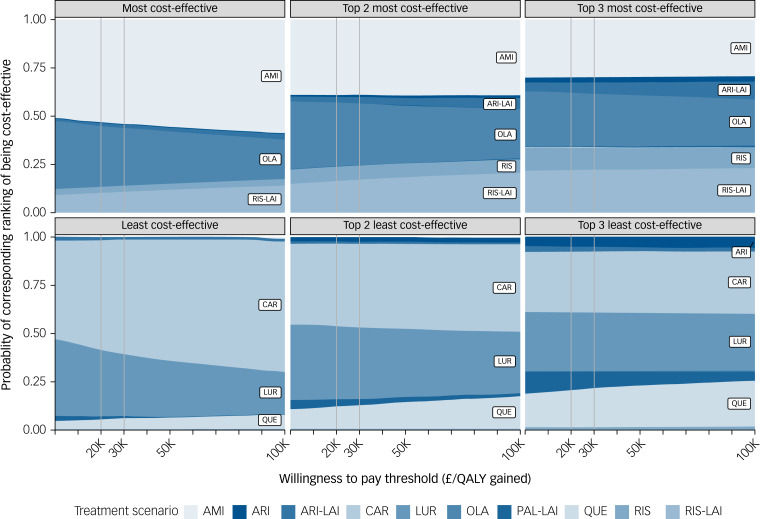
Probability of cost-effectiveness rankings among first-line antipsychotics. Treatment is an oral one if its name is not followed by ‘LAI’; AMI, amisulpride; ARI, aripiprazole; CAR, cariprazine; LAI, long-acting injection; LUR, lurasidone; OLA, olanzapine; PAL, paliperidone; QALY, quality-adjusted life year; QUE, quetiapine; RIS, risperidone.

In addition, amisulpride also led to the lowest costs (discounted: £147 197) compared to the others (£149 092–174 442), resulting in clear overall superiority (leading to higher QALYs and lower costs). At a WTP threshold of £20 000/QALY, the ranking from the most to the least cost-effective was amisulpride, olanzapine, risperidone-LAI, risperidone, aripiprazole-LAI, aripiprazole, paliperidone-LAI, quetiapine, lurasidone and cariprazine (Table 2, Supplementary Table 3). The ranking remained stable over WTPs from £0/QALY to £100 000/QALY (Supplementary Figure 1). Taking into account the uncertainty around all the model parameters, at a WTP threshold of £20 000/QALY, the most cost-effective first-line antipsychotics were amisulpride, olanzapine and risperidone-LAI with total probability of rankings of 1, ≤2 and ≤3, that is, 95%, 89% and 80% (amisulpride: 54%, 39%, 30%; olanzapine: 31%, 33%, 28%; risperidone-LAI: 10%, 17%, 22%), respectively; meanwhile, the least cost-effective were cariprazine, lurasidone and quetiapine with total probability of rankings of 10, ≥9 and ≥8, that is, 96%, 92% and 81% (cariprazine: 57%, 43%, 32%; lurasidone: 34%, 38%, 31%; quetiapine: 5%, 12%, 19%), respectively. The probabilities remained stable over WTPs from £0/QALY to £100 000/QALY (Fig. 2, Supplementary Figure 2).

The most influential parameters for cost-effectiveness were risk ratio of relapse of compared treatments, probability of medication switch after relapse and annual probability of discontinuation (Supplementary Figure 3). They were also the most influential parameters on incremental QALYs, with incremental QALYs additionally largely affected by treatment effects on fasting glucose and utility weight at relapse state of schizophrenia (Supplementary Figure 4). Scenario analyses found results to be robust across different timeframes (Supplementary Figure 5(a)) and discount rates (Supplementary Figure 5(b)). A longer timeframe and smaller discount rate favour the ranking for oral aripiprazole at a WTP higher than £30 000/QALY. Ranking among these antipsychotics did not change when they were used as second-line treatment for patients with non-first-episode schizophrenia (Supplementary Figure 6).

## Discussion

We compared the cost-effectiveness of ten commonly used antipsychotics in first-episode schizophrenia in the UK using a *de novo* DES model, which captured the features of the previously published models for such evaluation in different countries. We applied the model and ranked the available first-line antipsychotics in terms of QALYs (best to worse: amisulpride, risperidone-LAI, aripiprazole-LAI, olanzapine, paliperidone-LAI, risperidone, aripiprazole, lurasidone, quetiapine and cariprazine) and cost-effectiveness (best to worst: amisulpride, olanzapine, risperidone-LAI, risperidone, aripiprazole-LAI, aripiprazole, paliperidone-LAI, quetiapine and lurasidone and cariprazine) across a wide range of WTP thresholds in the UK. Amisulpride, olanzapine and risperidone-LAI were consistently the top three most cost-effective antipsychotics, while quetiapine, lurasidone and cariprazine were consistently the three least cost-effective antipsychotics. Cost-effectiveness results were primarily driven by relapse relevant parameters, particularly including the treatment effect on relapse prevention. The model was made open source allowing further adaptation for evaluation in the other contexts.

Substantial inconsistencies have been reported in the conclusions of published economic models for antipsychotics.^35^ This variability may be attributed to considerable differences in the number and types of antipsychotics assessed, as well as inconsistent methods adopted by various studies. Of the published models, the most similar study to our application was the study conducted by NICE in 2007 comparing first-line antipsychotics for relapse prevention in schizophrenia for their guideline.^3^ The NICE model reported similar magnitudes of predicted ten year outcomes, but different ranking of antipsychotics from the most to the least cost-effective: zotepine, olanzapine, paliperidone, haloperidol, aripiprazole, risperidone and amisulpride.^3^ There are several possible explanations for the difference in our results. First, we included different antipsychotics, because (a) zotepine is no longer available in the UK, (b) lurasidone, cariprazine and LAI atypicals were not available in 2007 and (c) haloperidol is a typical antipsychotic that is no longer commonly used.^19^ Second, the annual costs of oral atypicals have changed substantially since the previous analysis was conducted – ranging from £696 to £1036 in 2007 to £23–160 in 2020. Finally, our study has benefited from the inclusion of a large amount of recent evidence. For example, we used the 2022 NMA,^36^ including the study by Lecrubier et al,^37^ showing favourable efficacy of relapse prevention by amisulpride. This was not included in the NMA^3^ used in the previous model,^3^ leading to much more favourable cost-effectiveness results for amisulpride in our model (where it was ranked most effective) compared to the NICE model (where it was ranked least effective).

Our finding of amisulpride's overall superiority compared to other agents strengthens recommendations made by these prior meta-research studies – that it should be given strong consideration as a first-line agent in schizophrenia. While other authors have already highlighted its favourable profile in terms of tolerability and propensity for side-effects, as well as its intriguing efficacy for difficult-to-treat ‘negative’ psychotic symptoms,^38^ our analysis confirms a strong overall performance in terms of QALYs and cost-effectiveness. On the other hand, the relatively positive results for olanzapine may be somewhat surprising, given that it is widely considered among the most damaging agents for metabolic side-effects, and meta-research has failed to demonstrate any clear advantage in efficacy. However, it does benefit from being a less expensive agent with relatively low rates of discontinuation. In further scenario analysis, olanzapine-LAI, the product rarely used because of its increased monitoring requirement, was found to be more cost-effective than five commonly used antipsychotics (Supplementary Figure 7). Further research is needed to explore whether it worthy to encourage clinicians to prescribe more olanzapine-LAI instead of lesser cost-effective agents.

Our study has several methodological strengths. First, we used a DES model, which as a patient-level model allowed us to consider wide ranges of patient heterogeneity, and had no arbitrary restriction on the length of cycle for simulation purpose used in Markov models. Second, we included features not found in existing models, such as long-term cardiovascular events and diabetes. While a few models attempted to consider these events, they typically did so using direct treatment effects, of which there is no reliable evidence. Instead, we modelled the treatment effect on these outcomes via metabolic side-effects, given solid evidence on the difference in metabolic impact between antipsychotics and their association with long-term outcomes. Finally, we used recent evidence to inform our model construction, ensuring that our model reflected up-to-date knowledge.

Our study had some limitations. First, the base-case model used the US population-based Framingham equations^25–27^ to model cardiovascular events and diabetes. However, there is no cardio-metabolic policy model to model all these outcomes that is available for patients with schizophrenia in the UK. PRIMROSE^39^ was the most relevant UK model, predicting risk of cardiovascular disease for people with severe mental illness, but its focus was the risk at the time point of ten years rather than the risks over years making it unsuitable for our target model, which requires prediction of disease progression. This analysis has highlighted the need for future research to produce validated cardio-metabolic policy models and cost evaluations for schizophrenia population. Second, while the model was developed to reflect clinical practice, assumptions were made considering data availability and model complexity. Treatment switch owing to side-effects was modelled through the pathway of discontinuation–relapse–switch, although patients may switch treatment before relapse. We assumed metabolic side-effects would occur and disappear once the patients started and discontinued antipsychotics, respectively, although some metabolic profiles (e.g. BMI) may not return to their original levels shortly after treatment stopping.^40^ Therefore, while our analysis can provide some overarching principles to guide decision-making, clinician discretion should still be applied in individual cases. Third, we used participant data from the synthetic data-set simulated from summary-level data, which might not capture the variability of the target population. However, our results were relatively stable across wide ranges of sensitivity analyses.

Our results may inform decision-making by clinicians, patients and their carers by ranking the available antipsychotics based on QALYs – integrating the overall efficacy and side-effects of each agent. The differences between antipsychotics in QALYs gained mainly reside with their efficacy in relapse prevention and the metabolic side-effects, which lead to higher risk of long-term cardio-metabolic adverse events. In addition, our cost-effectiveness results could be used to guide the choice of antipsychotics to improve healthcare resource efficiency when clinicians and patients do not have a specific preference over certain attributes of treatments (e.g. avoidance of weight gain). Our model provides a good foundation for the model-based economic evaluation in schizophrenia. With the model being open source, it could be used by others for their economic evaluation. In addition, the model provides insight for future research, namely that a better estimation of the model parameters will improve the quality of the evaluation, thus increasing the need for further research, such as more solid evidence examining treatment effect on relapse prevention.

The use of our *de novo* core DES model to assess antipsychotic treatment of first-episode schizophrenia in the UK has shown that amisulpride may be particularly effective in terms of QALYs and cost-effectiveness. Other medications, such as risperidone-LAI, may also be prioritised where amisulpride is not appropriate because of specific patient factors. The rankings of treatment generated by our analysis could be used to optimise treatment algorithms for first-episode schizophrenia and facilitate informed patient choice and shared decision-making.

## Supporting information

Zhou et al. supplementary material Zhou et al. supplementary material

## Data Availability

All needed data and materials are presented in the manuscript or the supporting information. No extra data will be needed. The codes of the model were available at https://github.com/zhoujunwen/HE-DES-Schizophrenia-UK.
