# Supplementary material for: Cost-effectiveness of ten commonly used antipsychotics in first-episode schizophrenia in the UK: economic evaluation based on a de novo discrete event simulation model
Source: Br J Psychiatry. 2025 Aug;227(2):545–52. doi: 10.1192/bjp.2024.251 (PMC12355477; doi:10.1192/bjp.2024.251)
Supplement: Zhou et al. supplementary material [file S0007125024002514sup001.pdf]

Cost-effectiveness of ten commonly used antipsychotics in first-episode schizophrenia in the UK: economic evaluation based on a de-novo discrete event simulation model

Supplementary materials

Table of Contents

Supplementary sections 2
Supplementary section 1. Simulation of hypothetical cohorts for base-case and scenario analyses ... 2
Supplementary section 2. Model structure of previous economic models in schizophrenia 3
Decision tree model 3
Cohort-level Markov model 4
Patient-level Markov model 5
Discrete event simulation model 7
Supplementary section 3. Time to event simulation with an example of time to relapse simulation.. 9
Supplementary section 4. Illustration of the use of the DES model. 10
Supplementary tables 11
Supplementary table 1. Number of prescriptions of atypical antipsychotics in the UK over 2014-2022 11
Supplementary table 2. Model input for the base-case, DSA and PSA analyses 12
Supplementary table 3. Base-case incremental outcomes over 10 years under compared first-line antipsychotics 19
Supplementary table 4. Cost-effectiveness results across all the scenarios 20
Supplementary figures 22
Supplementary figure 1. Base-case rankings of cost-effectiveness among first-line antipsychotics 22
Supplementary figure 2. Probabilistic sensitivity analyses results of the ranking among comparators 23
Supplementary figure 3. Top 3 impactful inputs (n=263) on NMB at £20K/QALY in AMI vs. others 24
Supplementary figure 4. Top 3 impactful inputs (n=263) on incremental QALYs in AMI vs. others 25
Supplementary figure 5a. Scenario analyses results with different analytical timeframes 26
Supplementary figure 5b. Scenario analyses results with different discount rates 27
Supplementary figure 6. Scenario analyses results comparing antipsychotics as second-line treatment in non-first-episode schizophrenia 28
Supplementary figure 7. Scenario analyses results including olanzapine-LAI into the comparison among first-line antipsychotics in first-episode schizophrenia 29
Supplementary reference 30

Supplementary sections

Supplementary section 1. Simulation of hypothetical cohorts for base-case and scenario analyses

For the base-case analyses on patients with first-episode schizophrenia, a hypothetical cohort of 1000 participants were simulated primarily based on the summary characteristics of study population from Smith et al1, which assessed the metabolic profiles of the young people with first episode psychosis in the UK. For the scenario analyses on patients with non-first-episode schizophrenia, a hypothetical cohort of 1000 participants were simulated primarily based on the summary characteristics of study population from the STAR trial2, which compared aripiprazole with standard treatment (especially olanzapine) for people with schizophrenia. Other published studies on patients with schizophrenia and assumptions were used to support the simulation of the hypothetical cohorts (Table SS1). Each attribute of an individual was simulated independently based on the data type of the covariate: continuous covariates were simulated using the normal distribution based on the population mean and standard deviance; binary covariates were simulated using the Bernoulli distribution based on the population proportion; and categorical covariates were simulated using a generalized Bernoulli distribution based on the population proportion for each category.

Table SS1. Baseline characteristics of the target patients with schizophrenia for the simulation.

	First-episode schizophrenia cohort for base-case analyses		Non-first-episode schizophrenia cohort for the scenario analysis	
**Attributes**	**Mean (SD)/ Proportion**	**Source**	**Mean (SD)/ Proportion**	**Source**
**Age, years**	24.6 (3.9)	Smith 2020[1]	38.5 (10.9)	Barnett 2009[2]
**Male**	0.69	Smith 2020[1]	0.60	Barnett 2009[2]
**Body mass index, kg/m2**	27.8 (5.8)	Smith 2020[1]	27.2 (5.1)	Barnett 2009[2]
**Total cholesterol, mg/dl**	173.7 (34.7)	Smith 2020[1]	211.6 (42.5)	Barnett 2009[2]
**HDL cholesterol, mg/dl**	46.3 (11.6)	Smith 2020[1]	50.4 (15.4)	Barnett 2009[2]
**Triglycerides, mg/dl**	150.4 (97.3)	Smith 2020[1]	192.4 (141.6)	Barnett 2009[2]
**Fasting glucose, mg/dl**	90.1 (12.6)	Smith 2020[1]	97.4 (19.1)	Barnett 2009[2]
**Systolic blood pressure, mmHg**	125.8 (17.6)	Smith 2020[1]	131.7 (19.3)	Osborn 2015[3]
**Current smoker**	0.61	Smith 2020[1]	0.49	Osborn 2015[3]
**Alcohol consumption, unit/week**	1 (0)	Assumption	1 (0)	Assumption
**Compliance**				
Full	0.41	Gilmer 2004[4]	0.41	Gilmer 2004[4]
Partial	0.35	Gilmer 2004[4]	0.35	Gilmer 2004[4]
None	0.24	Gilmer 2004[4]	0.24	Gilmer 2004[4]
**Parental history of diabetes**	0	Assumption	0	Assumption
**Use of antihypertensive therapy**	0	Assumption	0.16	Smith 2013[5]
**History of atrial fibrillation**	0	Assumption	0.01	Smith 2013[5]
**History of left ventricular hypertrophy**	0	Assumption	0	Assumption
**History of diabetes**	0	Assumption	0.09	Smith 2013[5]
**History of coronary heart disease**	0	Assumption	0.06	Smith 2013[5]
**History of stroke**	0	Assumption	0.04	Smith 2013[5]

HDL, high density lipoprotein; SD, standard deviance

Supplementary section 2. Model structure of previous economic models in schizophrenia

We reviewed the model structure of the models for economic evaluation in schizophrenia[6-89], identified from previous systematic review[90] and an updated search in 2019[91]. We extracted information on the model structure, including branching points and branches for decision tree (DT) models, health states and transitions for cohort-level (CL) and patient-level (PL) Markov models (MM), and events for discrete event simulation (DES) model. Description analyses were performed on the extracted information for DT and CLMM models. For PLMM and DES model, the model structure of the original core model was described in detailed, since only two original core models were available in each of them. The difference between the adapted models and the original core model were summarized.

To compare the model structure in different types of model, the structural elements were grouped into pathway, which was used to represent the evolution of certain status. For example, assuming the pathway of schizophrenia disease progression is that, a stable patient may "relapse" and move to "Acute" status, then may "recover" and move back to the "Stable" status. In DT model, this can be represented by a branching point of "relapse?" followed by two branches of "no relapse" and "relapse", with "relapse" followed by another branching point of "remission?" further followed by two branches of "remission" and "no remission". In Markov model, this can be represented by the health states of "Stable" and "Acute", and the transition between them. In DES model, this can be represented by events of "Relapse" and "Remission", with both possible to happen after the other.

Decision tree model

Twenty-nine DT models (85.3% of all DT models) reported their model structure, evaluating 2 to 64 outcomes and 1 to 12 branching points with increasing model complexity. Most of them covered disease progression, treatment sequence and side effect. Most had branching points at relapse, response, discontinuation, and adherence. Other rare structures were applied, including different side effects, suicide and employability. The relapse branch was composed of stable and relapse, with around half of them further classifying relapse depending on the necessity of hospitalization. The response branch was composed of response and no response. The discontinuation branch was composed of continuation, discontinuation, with discontinuation sometimes followed by a switch or dose increase or discontinuing medication all together. The adherence branch was composed of adherent, non-adherent, and sometimes partially adherent. (Table SS1)

Table SS1. Branching points in the decision tree models

Pathway	Branching point	Level	Branch
**Disease progression**	Relapse (n=21)	2	• relapse, stable (or no relapse, relapse)<br>• exacerbation, stable<br>• hospitalization, stable
		3	• exacerbation, hospitalization, stable (or relapse requiring hospitalization, relapse not requiring hospitalization, stable)
	Response (n = 12)	2	• no response, response<br>• clinical deterioration, response<br>• inadequate response, response
**Treatment behaviour**	Discontinuation (n = 19)	2	• continue, discontinue<br>• continue, switch<br>• switch, discontinue medication all together
		3	• continue, increase dose, switch<br>• continue, switch, discontinue medication all together
		4	• continue, discontinue medication all together, switch due to side effect, switch due to lack of efficacy
	Adherence (n = 11)	2	• adherent, non-adherent
		3	• adherent, non-adherent, partially adherent
**Side effect**	Diabetes (n=2)	2	• no, yes
	metabolic syndrome (n=2)	2	• no, yes
	EPS (n=2)	2	• no, yes
	Therapy acceptability (n=1)	2	• no, yes
	Anticholinergic use (n=1)	2	• no, yes
	Diabetes complications (n=1)	7	• no complication, amputation, fatal MI, heart failure, ischemic heart disease, non-fatal MI, non-fatal stroke
	Side effect (n=1)	4	• no side effect, diabetes, EPS, intolerance glucose, weight gain
**Others**	Employability(n=1)	2	• no, yes
	Hospitalization (n=2)	2	• discharge from hospital, remain hospitalization
	Suicide (n=2)	2	• no suicide, suicide attempt/completion

EPS: extrapyramidal symptom; MI: myocardial infarction

Cohort-level Markov model

Thirty-one CLMM models (91% of all CLMM models) reported their structure, considering Markov health states from 2 to 33. Most of them reported the structure considering death and disease progression. Other pathways included presence of side effects, treatment lines, treatment status, setting of care, and adherence levels. Most models reported a structure with overall death, whereas others considered suicide specifically. There were seven ways of classification for schizophrenia health state, with 2-state classification of stable and relapse most used (Table SS2). Eight CLMM models did not include disease states, focused on the transition between the composite health states from parts of pathways of side effects, settings of care, treatment lines and treatment status.

Table SS2. Health states considered in the CLMM models

Pathway	Health states	N
**Disease progression**	schizophrenia, remission	2
	stable, relapse	14
	stable, non-stable, relapse	3
	no symptom, mix symptom, positive symptom, negative symptom, relapse	2
	residual, relapse	1
	PANSS improve >30%, PANSS improve 20%-30%, no response, post-relapse response, first relapse, subsequent relapse	2
	Mohr-Lenert health states 1-8	1
**Side effect (SE)**	no SE, SE	3
	no irreversible SE, irreversible SE	1
	no EPS, EPS	2
	no SE, tardive dyskinesia, agranulocytosis	2
	no SE, myocardial infarction, stroke	1
	no SE, metabolic syndrome, diabetes, coronary heart disease	1
	no SE, diabetes, stroke, CHD, hypertension, comorbidities (n=2,3,4)	1
**Treatment sequence**	comparator, clozapine	1
	≥ 2 lines of treatments	3
	3 lines of treatments	3
	4 lines of treatments	3
**Treatment status**	Treated, untreated	6
**Setting of care**	inpatient, outpatient	8
	hospitalization, outpatient intensive care, outpatient mild care	1
**Adherence**	adherent, non-adherent	3
	adherent, partially adherent, non-adherent	2
**Survival**	death, alive	21
	suicide, no suicide	5

CHD: coronary heart disease; EPS: extrapyramidal symptom; PANSS: positive and negative symptom scale; SE: side effect

Patient-level Markov model

Among PLMM models, two were original[52,76], whereas the others adapted the model by Furiak et al.[52].

<u>*Core PLMM model by Vera-Llonch et al*</u>[76]

Figure SS1 shows the recreated structure for the model by Vera-Llonch et al[76]. This model focused on treatment side effects. It considered the transition among six health states including "No side effect", "Prolactin-related disorder only", "Diabetes only", "Both prolactin-related disorder and diabetes", "Discontinuation" and "Death" at monthly cycle over 1-year timeframe. Patients differed in baseline weight and body mass index (BMI), with weight changed depending on baseline BMI, treatment taken and months on treatment. A weight change >5 kg might lead to treatment discontinuation.

Figure SS1. Recreated model structure for the PLMM model by Vera-Llonch et al

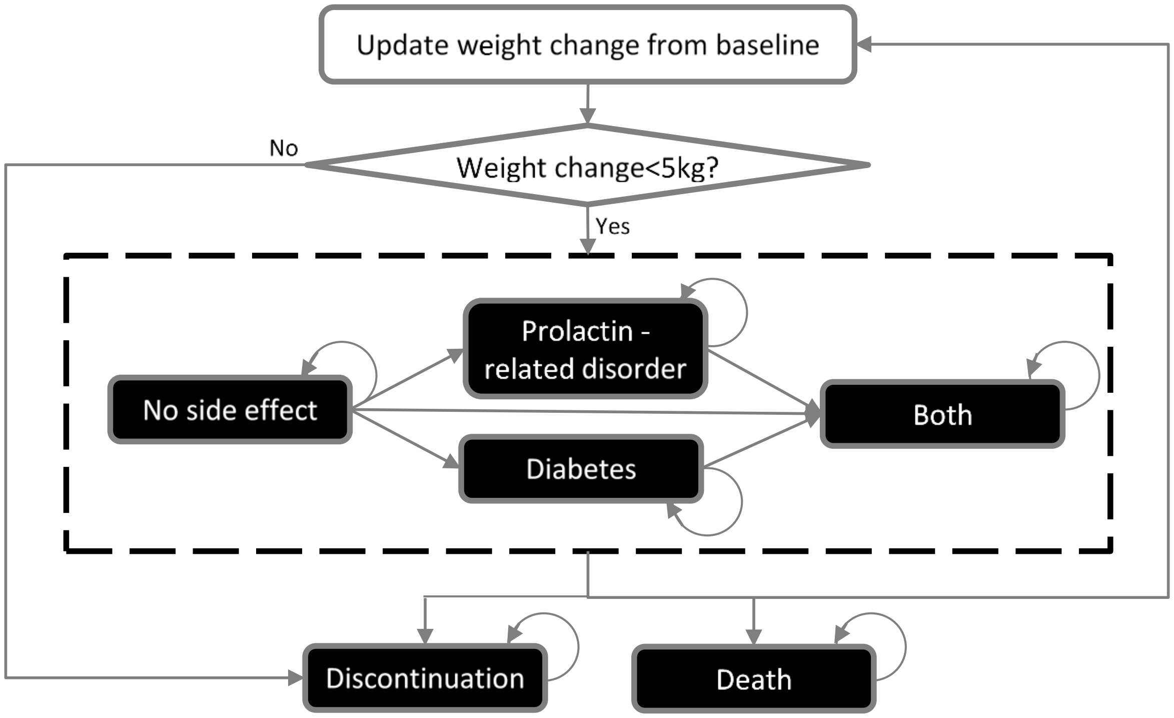

Core PLMM model by Furiak et al[52]

Figure SS2 shows the recreated structure for the model by Furiak et al[52] . This model covered the pathways of disease progression, side effects and treatment discontinuation, and modelled at 3-month cycle over 1-year timeframe. For disease progression, there were three health states: stable, relapse requiring or not requiring hospitalization. The side effect (SE) presented in the structure included weight gain, EPS, diabetes and hyperlipidaemia, each with 2 health states (with and without SE). Treatment discontinuation included continuation, discontinuing medication all together, and switch to the next line of treatment. Patients differed from each other in adherence level in baseline. As the cycle advanced, patients would have different risk of relapse either requiring or not requiring hospitalization, with varying number of previous relapses, adherence levels, treatments status. Patients would also have different probability of treatment switch and discontinuation depending on current state in schizophrenia, SE, and treatment status.

Figure SS2. Recreated model structure of the PLMM model by Furiak et al

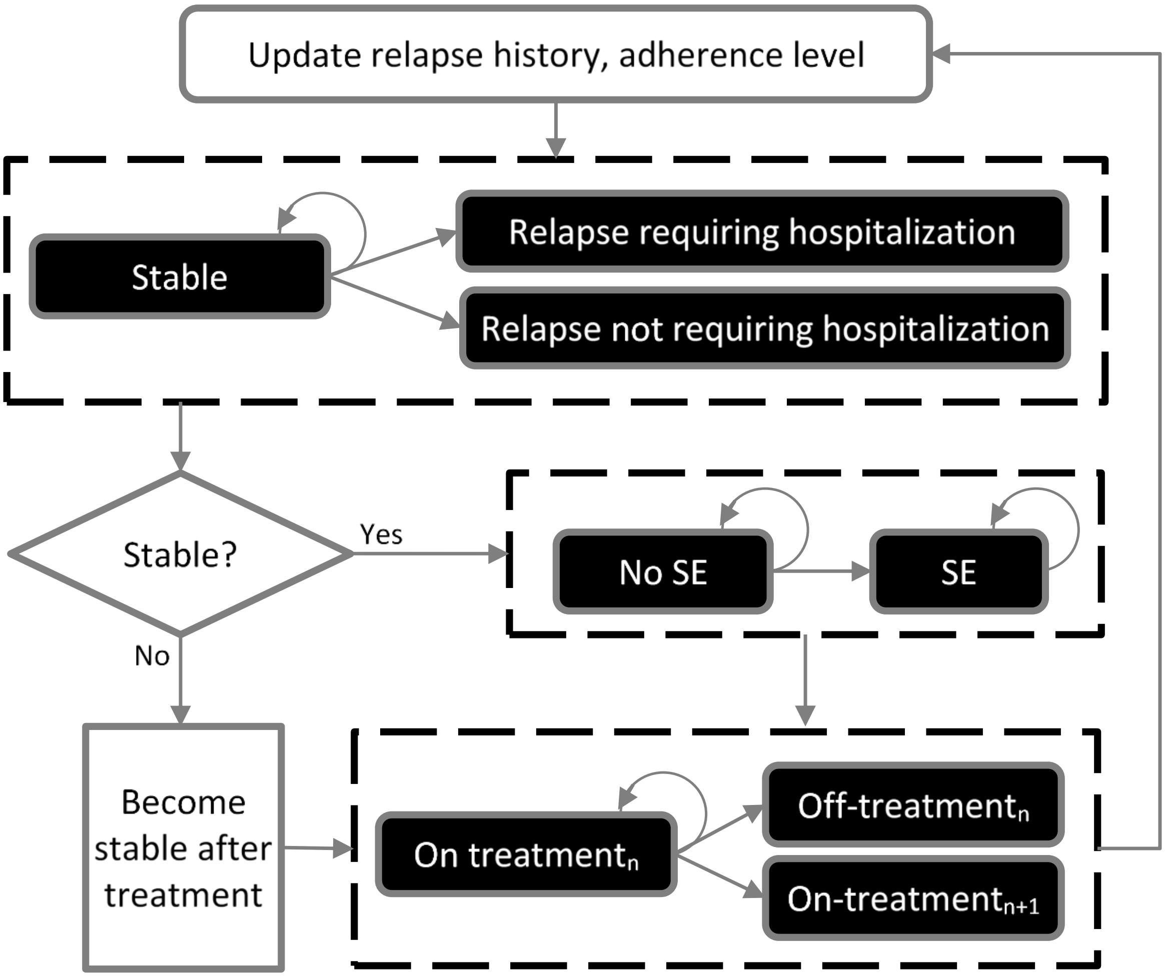

SE: side effect. The SE shown in the structure only included irreversible SE within the 1-year timeframe, including weight gain, EPS, diabetes, hyperlipidaemia.

Other non-core PLMM model

The other models[6,41,46] adapted the model structure by Furiak et al[52] with very limited changes in the model structure. Another model by Furiak et al[46] made a minor structural change, adding hyperprolactinemia and tardive dyskinesia as irreversible side effects. The others basically changed the model input due to the change of compared treatments and the change of country of analysis in their models.

Discrete event simulation model

Among DES models, two were original[25,70], whereas the others adapted the model by Heeg et al.[70].

Core DES model by Dilla et al[20]

Figure SS3 shows the recreated structure for the model by Dilla et al. This model covered the pathways of disease progression, side effect and treatment status. Relapse was reported as the only event in the pathway of disease progression, though remission should be also included in the model. Presence of side effects was also reported as the only event in the pathway of side effect, with side effects possibly considered as irreversible. In the pathway of treatment status, patients were possible to discontinue, switch or return to original treatments after relapse or side effects. Time seems advanced with relapse and the presence of side effects. Events in treatment status possibly happened right after relapse or presence of side effect, and remission possibly happened after certain period or ratio of time from the most recent relapse. Patients differ from each other mainly on the time spent on the health states in each pathway.

Figure SS3. Recreated model structure for the DES model by Dilla et al

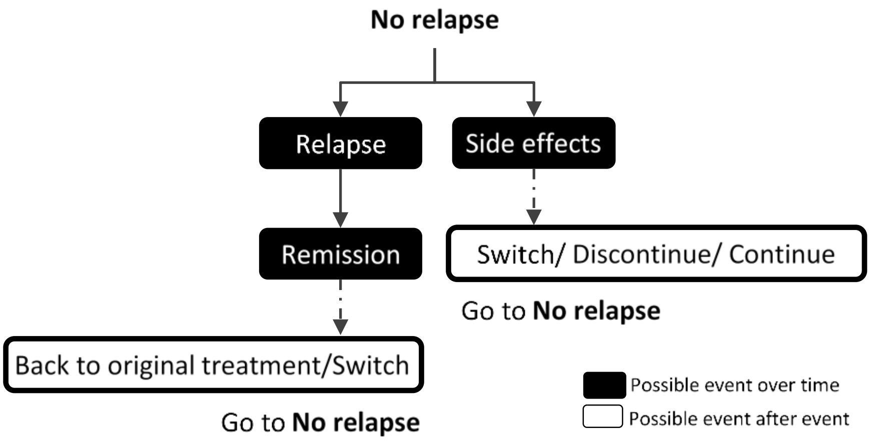

Core DES model by Heeg et al[70]

Figure SS4 shows the recreated structure for the model by Heeg et al[70]. This model covered multiple pathways, including disease progression, psychiatrist visit, treatment sequence, compliance, symptom score, patient care setting and ability of taking care of themselves. In disease progression pathway, patients may relapse from remission, or remission from relapse. In psychiatrist visit pathway, patients would move forward from the first to the next several visits after relapse and back to the first visit after relapse when they visit the psychiatrist after relapse. The model time advanced by time to remission, relapse and psychiatrist visit, and ended at the target timeframe or death. The transition in the other pathways happened right after these events. Patients differed from each other initially in the following characteristics: disease severity; risk of self-harming or harming others; possibility of side effect; and social and environmental factors with impact on their treatment location.

Figure SS4. Recreated model structure for the DES model by Heeg et al

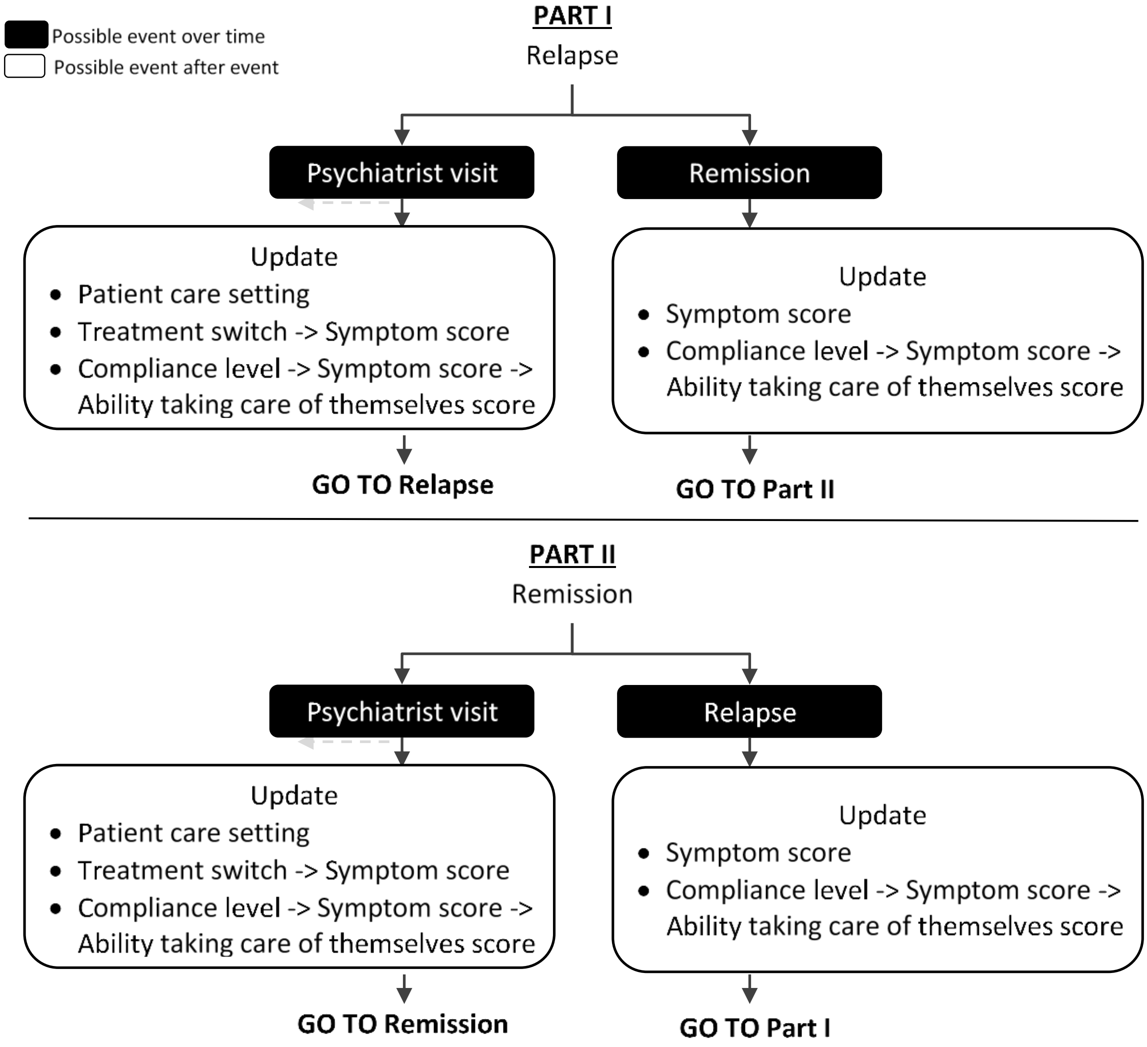

The other model[34,48,50,53,54,58,68,74] adapted the model by Heeg et al[70], with very limited changes in the model structure. Another model by Heeg et al[54] made a minor structural change, adding another compliance level and another line of treatment for switch. The others basically changed the model input due to the change of compared treatments (original: typical antipsychotic treatment with different compliance levels; adaptation: risperidone long-acting injection vs. others, branded risperidone vs. generic risperidone, different atypical antipsychotics, paliperidone extended release vs. others) and the change of country of analysis (original: the UK; adaptation: Canada, Germany, Spain, and Sweden) in their models.

Supplementary section 3. Time to event simulation with an example of time to relapse simulation.

To simulate a time to event for an individual, we

- firstly determined the time to event equation based on the survival equation reflecting the cumulative probability of no event ($S(t)$) over time (t) and the individual characteristics
- secondly generated a random number (θ) between 0 and 1 assuming it is the random probability of the event happening $S(t_\theta)$ at a particular time (t_θ)
- last derived the event time by identifying the corresponding time at the random probability of the event happening based on the time to event equation for this individual

Figure SS illustrate the above process. Take the simulation of time to relapse as an example, we

- firstly determined the time to relapse equation of the individual
    - Time to relapse was assumed following exponential distribution, therefore given the characteristics of an individual the rate (r) of relapse over time (t)is constant
    - The cumulative probability of no relapse $S(t) = \exp^{-rt}$
    - The above equation could be converted to: $t = \frac{-\ln(S(t))}{r}$
- secondly drew a random number (θ) between 0 and 1, assuming it is the random cumulative probability of no relapse $S(t_\theta)$
- last derived the time to relapse for this individual $t_\theta = \frac{-\ln(\theta)}{r}$

Figure SS. Illustrative example of time to event simulation.

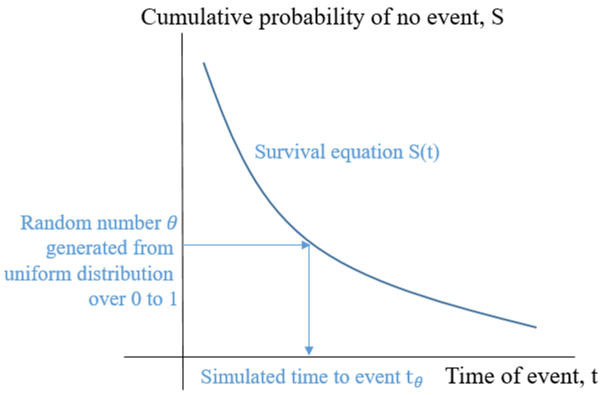

Supplementary section 4. Illustration of the use of the DES model.

To facilitate the use of the DES model, an RShiny webpage was developed with a case application to illustrate its use (available at https://livedataoxford.shinyapps.io/shiny_des_schizophrenia/), allowing users to perform economic evaluation of antipsychotics by modifying the treatment profiles and filling the data of a patient or a cohort of patients. The default patient was a 25-year old man with schizophrenia remitting from their first episode, being a non-smoker, not consuming any alcohol, partially compliant to antipsychotics, with the following metabolic profiles (BMI: 25 kg/m^2; total cholesterol: 150 mg/dl; HDL cholesterol: 40 mg/dl; Triglyceride: 140 mg/dl; fasting glucose: 80 mg/dl; systolic blood pressure: 120 mmHg), and without any following treatment or disease histories: use of antihypertensive treatment, atrial fibrillation, left ventricular hypertrophy, CHD, stroke, diabetes and parental diabetes. The default comparison was olanzapine compared with risperidone-LAI both as second-line treatment following first-line amisulpride, which were the undominated scenarios from the 90 treatment sequences in the base-case analyses (Table S4). Compared with olanzapine scenario, over ten years, risperidone-LAI scenario led to both higher QALYs (0·009) and higher costs (£2629), with an ICER of £298,465/QALY and net monetary benefit of £-2452 at a WTP of £20,000/QALY, indicating risperidone-LAI scenario was not cost-effective for this person.

Supplementary tables

Supplementary table 1. Number of prescriptions of atypical antipsychotics in the UK over 2014-2022

Atypical	2014	2015	2016	2017	2018	2019	2020	2021	2022
**Oral product**									
**Amisulpride**	396,244	401,170	405,509	403,363	403,682	403,457	409,740	399,643	396,371
**Aripiprazole**	721,923	820,177	925,007	1,028,029	1,131,199	1,252,622	1,369,540	1,484,867	1,572,613
**Cariprazine**	NA	NA	NA	NA	5	272	1,188	1,943	2,855
**Lurasidone**	91	2,702	6,975	12,759	16,581	21,230	26,769	32,044	37,748
**Olanzapine**	2,171,475	2,255,063	2,345,511	2,398,850	2,445,795	2,489,579	2,564,452	2,599,771	2,607,892
**Paliperidone[a]**	1,277	1,630	1,644	1,927	2,123	2,408	2,839	3,450	3,685
**Quetiapine**	2,577,302	2,811,606	3,082,370	3,322,969	3,517,655	3,735,141	4,013,204	4,200,612	4,315,352
**Risperidone**	1,621,477	1,682,245	1,722,382	1,744,097	1,761,325	1,766,177	1,784,804	1,772,142	1,772,200
**Long acting injection (LAI) product**									
**Aripiprazole**	NA	3,116	5,162	7,977	10,535	12,580	14,565	15,359	15,127
**Olanzapine[a]**	128	180	204	256	251	385	451	489	567
**Paliperidone**	5,451	7,823	9,828	11,520	12,336	13,349	14,314	14,738	14,830
**Risperidone**	28,266	25,185	22,469	19,747	17,314	15,052	12,976	10,973	9,647

Data obtained from Prescription Cost Analysis (PCA) – England database[92]

[a]: Oral paliperidone and depot olanzapine were not included in the present study as they were not commonly used in the current practice though they have been available since 2014. NA: not available;

Supplementary table 2. Model input for the base-case, DSA and PSA analyses

Model parameter	Base-case	DSA parameter		PSA parameter			Source
		Low	High	Distribution	Alpha	Beta	
**Annual probability of relapse**	0.72	0.65	0.77	Beta	137.55	54.17	Schneider-Thoma 2022[93]
**Risk ratio of relapse by treatment: amisulpride oral vs. placebo**	0.19	0.10	0.39	Lognorm	-1.65	0.36	Ostuzzi 2022[94], Schneider-Thoma 2022[93]
**Risk ratio of relapse by treatment: aripiprazole oral vs. placebo**	0.32	0.14	0.56	Lognorm	-1.14	0.35	Schneider-Thoma 2022[93]
**Risk ratio of relapse by treatment: cariprazine oral vs. placebo**	0.65	0.16	1.14	Lognorm	-0.43	0.50	Schneider-Thoma 2022[93]
**Risk ratio of relapse by treatment: lurasidone oral vs. placebo**	0.63	0.25	1.02	Lognorm	-0.46	0.36	Schneider-Thoma 2022[93]
**Risk ratio of relapse by treatment: olanzapine oral vs. placebo**	0.20	0.09	0.38	Lognorm	-1.61	0.37	Schneider-Thoma 2022[93]
**Risk ratio of relapse by treatment: quetiapine oral vs. placebo**	0.47	0.24	0.72	Lognorm	-0.76	0.28	Schneider-Thoma 2022[93]
**Risk ratio of relapse by treatment: risperidone oral vs. placebo**	0.29	0.14	0.49	Lognorm	-1.24	0.32	Schneider-Thoma 2022[93]
**Risk ratio of relapse by treatment: clozapine oral vs. placebo**	0.06	0.03	0.11	Lognorm	-2.82	0.30	Essali 2009[95], Schneider-Thoma 2022[93]
**Risk ratio of relapse by treatment: aripiprazole LAI vs. placebo**	0.32	0.14	0.57	Lognorm	-1.14	0.36	Schneider-Thoma 2022[93]
**Risk ratio of relapse by treatment: paliperidone LAI vs. placebo**	0.31	0.16	0.48	Lognorm	-1.17	0.28	Schneider-Thoma 2022[93]
**Risk ratio of relapse by treatment: risperidone LAI vs. placebo**	0.25	0.12	0.47	Lognorm	-1.39	0.35	Schneider-Thoma 2022[93]
**Hazard ratio of relapse by compliance: partial vs. full**	1.79	1.31	2.43	Lognorm	0.58	0.16	Gilmer 2004[4]
**Hazard ratio of relapse by compliance: none vs. full**	2.59	1.88	3.56	Lognorm	0.95	0.16	Gilmer 2004[4]
**Duration of relapse, years**	0.50	0.38	0.63	Norm	0.50	0.06	NICE 2014[96] (Range: ±25%)
**Annual probability of discontinuation using amisulpride oral**	0.25	0.16	0.38	Beta	13.71	41.72	Ostuzzi 2022[94], Schneider-Thoma 2022[93]
**Annual probability of discontinuation using aripiprazole oral**	0.46	0.33	0.58	Beta	27.02	31.85	Schneider-Thoma 2022[93]
**Annual probability of discontinuation using cariprazine oral**	0.61	0.35	0.82	Beta	9.64	6.11	Schneider-Thoma 2022[93]
**Annual probability of discontinuation using lurasidone oral**	0.51	0.35	0.67	Beta	19.49	18.54	Schneider-Thoma 2022[93]
**Annual probability of discontinuation using olanzapine oral**	0.27	0.18	0.36	Beta	25.79	70.53	Schneider-Thoma 2022[93]
**Annual probability of discontinuation using quetiapine oral**	0.42	0.31	0.54	Beta	27.60	38.00	Schneider-Thoma 2022[93]
**Annual probability of discontinuation using risperidone oral**	0.31	0.21	0.41	Beta	24.94	56.56	Schneider-Thoma 2022[93]
**Annual probability of discontinuation using clozapine oral**	0.33	0.22	0.49	Beta	14.96	30.64	Kishimoto 2019[97], Schneider-Thoma 2022[93]
**Annual probability of discontinuation using aripiprazole LAI**	0.36	0.26	0.47	Beta	27.37	48.76	Schneider-Thoma 2022[93]
**Annual probability of discontinuation using paliperidone LAI**	0.43	0.33	0.51	Beta	47.39	63.24	Schneider-Thoma 2022[93]
**Annual probability of discontinuation using risperidone LAI**	0.34	0.23	0.47	Beta	19.58	37.31	Schneider-Thoma 2022[93]
**Proportion of switch after relapse**	0.50	0.25	0.75	Beta	7.18	7.18	Assumption
**Proportion of acute EPS using amisulpride oral**	0.28	0.18	0.38	Beta	20.78	52.76	Leucht 2013[98], Schneider-Thoma 2022[93]
**Proportion of acute EPS using aripiprazole oral**	0.41	0.22	0.59	Beta	11.17	15.81	Schneider-Thoma 2022[93]
**Proportion of acute EPS using cariprazine oral**	0.36	0.15	0.57	Beta	6.61	11.89	Schneider-Thoma 2022[93]
**Proportion of acute EPS using lurasidone oral**	0.19	0.09	0.44	Beta	3.43	14.73	Schneider-Thoma 2022[93]
**Proportion of acute EPS using olanzapine oral**	0.04	0.01	0.23	Beta	0.52	11.56	Schneider-Thoma 2022[93]
**Proportion of acute EPS using quetiapine oral**	0.21	0.12	0.39	Beta	7.49	27.93	Schneider-Thoma 2022[93]
**Proportion of acute EPS using risperidone oral**	0.26	0.13	0.48	Beta	6.15	17.67	Schneider-Thoma 2022[93]
**Proportion of acute EPS using clozapine oral**	0.06	0.02	0.13	Beta	5.11	73.58	Leucht 2013[98], Schneider-Thoma 2022[93]
**Proportion of acute EPS using aripiprazole LAI**	0.45	0.26	0.62	Beta	13.23	16.08	Schneider-Thoma 2022[93]
**Proportion of acute EPS using paliperidone LAI**	0.35	0.23	0.53	Beta	13.16	24.42	Schneider-Thoma 2022[93]
**Proportion of acute EPS using risperidone LAI**	0.27	0.14	0.53	Beta	4.98	13.41	Schneider-Thoma 2022[93]
**Proportion of weight gain using amisulpride oral**	0.27	0.13	0.45	Beta	7.84	21.08	NICE 2014[96]
**Proportion of weight gain using aripiprazole oral**	0.13	0.07	0.22	Beta	9.29	63.00	NICE 2014[96]
**Proportion of weight gain using cariprazine oral**	0.21	0.06	0.71	Beta	1.00	3.87	Ostuzzi 2022[94], NICE 2014[96]
**Proportion of weight gain using lurasidone oral**	0.19	0.05	0.67	Beta	0.99	4.26	Ostuzzi 2022[94], NICE 2014[96]
**Proportion of weight gain using olanzapine oral**	0.36	0.25	0.47	Beta	26.43	46.15	NICE 2014[96]
**Proportion of weight gain using quetiapine oral**	0.40	0.13	1.00	Beta	1.53	2.30	Ostuzzi 2022[94], NICE 2014[96]

Model parameter	Base-case	DSA parameter		PSA parameter			Source
		Low	High	Distribution	Alpha	Beta	
**Proportion of weight gain using risperidone oral**	0.18	0.09	0.29	Beta	9.93	45.59	NICE 2014[96]
**Proportion of weight gain using clozapine oral**	0.26	0.21	0.31	Beta	88.27	256.52	Essali 2009[95], NICE 2014[96]
**Proportion of weight gain using aripiprazole LAI**	0.13	0.07	0.22	Beta	9.29	63.00	NICE 2014[96]
**Proportion of weight gain using paliperidone LAI**	0.18	0.08	0.30	Beta	7.96	36.90	NICE 2014[96]
**Proportion of weight gain using risperidone LAI**	0.18	0.09	0.29	Beta	9.93	45.59	NICE 2014[96]
**Proportion of sedation using amisulpride oral**	0.03	0.01	0.05	Beta	10.07	347.52	Leucht 2013[98], Schneider-Thoma 2022[93]
**Proportion of sedation using aripiprazole oral**	0.01	0.01	0.03	Beta	7.41	521.88	Schneider-Thoma 2022[93]
**Proportion of sedation using cariprazine oral**	0.01	0.00	0.02	Beta	3.57	353.91	Ostuzzi 2022[94], Schneider-Thoma 2022[93]
**Proportion of sedation using lurasidone oral**	0.02	0.01	0.05	Beta	4.81	194.14	Schneider-Thoma 2022[93]
**Proportion of sedation using olanzapine oral**	0.03	0.02	0.05	Beta	11.54	378.42	Schneider-Thoma 2022[93]
**Proportion of sedation using quetiapine oral**	0.06	0.04	0.09	Beta	15.90	267.98	Schneider-Thoma 2022[93]
**Proportion of sedation using risperidone oral**	0.02	0.01	0.04	Beta	7.94	328.37	Schneider-Thoma 2022[93]
**Proportion of sedation using clozapine oral**	0.15	0.09	0.24	Beta	13.83	76.82	Leucht 2013[98], Schneider-Thoma 2022[93]
**Proportion of sedation using aripiprazole LAI**	0.04	0.01	0.12	Beta	2.42	53.36	Schneider-Thoma 2022[93]
**Proportion of sedation using paliperidone LAI**	0.04	0.02	0.08	Beta	5.15	141.94	Schneider-Thoma 2022[93]
**Proportion of sedation using risperidone LAI**	0.01	0.01	0.03	Beta	5.13	341.16	Schneider-Thoma 2022[93]
**Proportion of sexual dysfunction using amisulpride oral**	0.03	0.01	0.07	Beta	3.00	106.00	Schmidt-Kraepelin 2022[99], Serretti 2011[100]
**Proportion of sexual dysfunction using aripiprazole oral**	0.27	0.17	0.39	Beta	17.00	45.00	Serretti 2011[100]
**Proportion of sexual dysfunction using cariprazine oral**	0.01	0.01	0.01	Beta	20	2028	Schmidt-Kraepelin 2022[99], Serretti 2011[100]
**Proportion of sexual dysfunction using lurasidone oral**	0.00	NA	NA	NA	NA	NA	Clayton 2018[101]
**Proportion of sexual dysfunction using olanzapine oral**	0.40	0.39	0.42	Beta	1421	2100	Serretti 2011[100]
**Proportion of sexual dysfunction using quetiapine oral**	0.15	0.13	0.17	Beta	213	1233	Serretti 2011[100]
**Proportion of sexual dysfunction using risperidone oral**	0.45	0.43	0.48	Beta	864	1038	Serretti 2011[100]
**Proportion of sexual dysfunction using clozapine oral**	0.53	0.43	0.62	Beta	58.00	52.00	Serretti 2011[100]
**Proportion of sexual dysfunction using aripiprazole LAI**	0.27	0.17	0.39	Beta	17.00	45.00	Serretti 2011[100]
**Proportion of sexual dysfunction using paliperidone LAI**	0.57	0.38	0.73	Beta	17.16	13.17	Potkin 2017[102], Serretti 2011[100]
**Proportion of sexual dysfunction using risperidone LAI**	0.45	0.43	0.48	Beta	864	1038	Serretti 2011[100]
**Annual probability of tardive dyskinesia using amisulpride oral**	0.02	0.00	0.05	Beta	3.26	134.30	Carbon 2018[103]
**Annual probability of tardive dyskinesia using aripiprazole oral**	0.02	0.00	0.04	Beta	2.64	154.16	Carbon 2018[103]
**Annual probability of tardive dyskinesia using cariprazine oral**	0.05	0.00	0.09	Beta	4.03	82.00	Assume = Lurasidone
**Annual probability of tardive dyskinesia using lurasidone oral**	0.05	0.00	0.09	Beta	4.03	82.00	Carbon 2018[103]
**Annual probability of tardive dyskinesia using olanzapine oral**	0.03	0.02	0.04	Beta	29.25	993.97	Carbon 2018[103]
**Annual probability of tardive dyskinesia using quetiapine oral**	0.02	0.00	0.05	Beta	4.51	178.29	Carbon 2018[103]
**Annual probability of tardive dyskinesia using risperidone oral**	0.02	0.01	0.03	Beta	16.69	687.16	Carbon 2018[103]
**Annual probability of tardive dyskinesia using clozapine oral**	0.04	0.02	0.06	Beta	10.80	251.80	Carbon 2018[103]
**Annual probability of tardive dyskinesia using aripiprazole LAI**	0.02	0.00	0.04	Beta	2.64	154.16	Carbon 2018[103]
**Annual probability of tardive dyskinesia using paliperidone LAI**	0.02	0.01	0.03	Beta	16.69	687.16	Assume = Risperidone
**Annual probability of tardive dyskinesia using risperidone LAI**	0.02	0.01	0.03	Beta	16.69	687.16	Carbon 2018[103]
**Proportion of agranulocytosis using clozapine**	0.01	0.01	0.01	Beta	93	12667	Munro 1999[104]
**Mean difference of BMI (kg/m2) by treatment: amisulpride oral vs. placebo**	1.66	0.18	3.14	Norm	1.66	0.76	Deepak 2015[105]
**Mean difference of BMI (kg/m2) by treatment: aripiprazole oral vs. placebo**	-0.22	-0.81	0.37	Norm	-0.22	0.30	Pillinger 2020[106]
**Mean difference of BMI (kg/m2) by treatment: cariprazine oral vs. placebo**	0.83	0.00	1.66	Norm	0.83	0.42	Greger 2021[107]
**Mean difference of BMI (kg/m2) by treatment: lurasidone oral vs. placebo**	0.24	0.08	0.41	Norm	0.24	0.08	Pillinger 2020[106]
**Mean difference of BMI (kg/m2) by treatment: olanzapine oral vs. placebo**	1.07	0.90	1.25	Norm	1.07	0.09	Pillinger 2020[106]
**Mean difference of BMI (kg/m2) by treatment: quetiapine oral vs. placebo**	0.70	0.44	0.96	Norm	0.70	0.13	Pillinger 2020[106]

Model parameter	Base-case	DSA parameter		PSA parameter			Source
		Low	High	Distribution	Alpha	Beta	
**Mean difference of BMI (kg/m2) by treatment: risperidone oral vs. placebo**	0.56	0.42	0.70	Norm	0.56	0.07	Pillinger 2020[106]
**Mean difference of BMI (kg/m2) by treatment: clozapine oral vs. placebo**	1.02	0.27	1.78	Norm	1.02	0.39	Pillinger 2020[106]
**Mean difference of BMI (kg/m2) by treatment: aripiprazole LAI vs. placebo**	-0.22	-0.81	0.37	Norm	-0.22	0.30	Pillinger 2020[106]
**Mean difference of BMI (kg/m2) by treatment: paliperidone LAI vs. placebo**	0.56	0.42	0.70	Norm	0.56	0.07	Pillinger 2020[106]
**Mean difference of BMI (kg/m2) by treatment: risperidone LAI vs. placebo**	0.56	0.42	0.70	Norm	0.56	0.07	Pillinger 2020[106]
**Mean difference of total cholesterol (mg/dL) by treatment: amisulpride oral vs. placebo**	8.11	-13.90	30.12	Norm	8.11	11.23	Pillinger 2020[106]
**Mean difference of total cholesterol (mg/dL) by treatment: aripiprazole oral vs. placebo**	2.32	-1.93	5.79	Norm	2.32	1.97	Pillinger 2020[106]
**Mean difference of total cholesterol (mg/dL) by treatment: cariprazine oral vs. placebo**	-3.47	-9.27	2.70	Norm	-3.47	3.05	Pillinger 2020[106]
**Mean difference of total cholesterol (mg/dL) by treatment: lurasidone oral vs. placebo**	-1.16	-0.58	3.47	Norm	-1.16	1.03	Pillinger 2020[106]
**Mean difference of total cholesterol (mg/dL) by treatment: olanzapine oral vs. placebo**	15.44	11.97	18.92	Norm	15.44	1.77	Pillinger 2020[106]
**Mean difference of total cholesterol (mg/dL) by treatment: quetiapine oral vs. placebo**	11.97	7.34	16.22	Norm	11.97	2.27	Pillinger 2020[106]
**Mean difference of total cholesterol (mg/dL) by treatment: risperidone oral vs. placebo**	2.32	-1.16	5.79	Norm	2.32	1.77	Pillinger 2020[106]
**Mean difference of total cholesterol (mg/dL) by treatment: clozapine oral vs. placebo**	21.62	10.04	33.20	Norm	21.62	5.91	Pillinger 2020[106]
**Mean difference of total cholesterol (mg/dL) by treatment: aripiprazole LAI vs. placebo**	2.32	-1.93	5.79	Norm	2.32	1.97	Pillinger 2020[106]
**Mean difference of total cholesterol (mg/dL) by treatment: paliperidone LAI vs. placebo**	2.32	-1.16	5.79	Norm	2.32	1.77	Pillinger 2020[106]
**Mean difference of total cholesterol (mg/dL) by treatment: risperidone LAI vs. placebo**	2.32	-1.16	5.79	Norm	2.32	1.77	Pillinger 2020[106]
**Mean difference of HDL cholesterol (mg/dL) by treatment: amisulpride oral vs. placebo**	-3.86	-12.74	5.41	Norm	-3.86	4.63	Pillinger 2020[106]
**Mean difference of HDL cholesterol (mg/dL) by treatment: aripiprazole oral vs. placebo**	1.54	0.00	3.09	Norm	1.54	0.79	Pillinger 2020[106]
**Mean difference of HDL cholesterol (mg/dL) by treatment: cariprazine oral vs. placebo**	0.77	-1.54	3.09	Norm	0.77	1.18	Pillinger 2020[106]
**Mean difference of HDL cholesterol (mg/dL) by treatment: lurasidone oral vs. placebo**	0.77	-1.93	3.47	Norm	0.77	1.38	Pillinger 2020[106]
**Mean difference of HDL cholesterol (mg/dL) by treatment: olanzapine oral vs. placebo**	-0.39	-1.54	1.16	Norm	-0.39	0.69	Pillinger 2020[106]
**Mean difference of HDL cholesterol (mg/dL) by treatment: quetiapine oral vs. placebo**	0.39	-1.16	1.93	Norm	0.39	0.79	Pillinger 2020[106]
**Mean difference of HDL cholesterol (mg/dL) by treatment: risperidone oral vs. placebo**	0.39	-0.77	1.93	Norm	0.39	0.69	Pillinger 2020[106]
**Mean difference of HDL cholesterol (mg/dL) by treatment: clozapine oral vs. placebo**	-4.48	-6.81	-2.15	Norm	-4.48	1.19	Gupta 2014[108]
**Mean difference of HDL cholesterol (mg/dL) by treatment: aripiprazole LAI vs. placebo**	1.54	0.00	3.09	Norm	1.54	0.79	Pillinger 2020[106]
**Mean difference of HDL cholesterol (mg/dL) by treatment: paliperidone LAI vs. placebo**	0.39	-0.77	1.93	Norm	0.39	0.69	Pillinger 2020[106]
**Mean difference of HDL cholesterol (mg/dL) by treatment: risperidone LAI vs. placebo**	0.39	-0.77	1.93	Norm	0.39	0.69	Pillinger 2020[106]
**Mean difference of triglycerides (mg/dL) by treatment: amisulpride oral vs. placebo**	7.96	-44.25	60.18	Norm	7.96	26.64	Pillinger 2020[106]
**Mean difference of triglycerides (mg/dL) by treatment: aripiprazole oral vs. placebo**	1.77	-6.19	9.73	Norm	1.77	4.06	Pillinger 2020[106]
**Mean difference of triglycerides (mg/dL) by treatment: cariprazine oral vs. placebo**	0.88	-11.50	12.39	Norm	0.88	6.10	Pillinger 2020[106]
**Mean difference of triglycerides (mg/dL) by treatment: lurasidone oral vs. placebo**	0.00	-12.39	11.50	Norm	0.00	6.10	Pillinger 2020[106]
**Mean difference of triglycerides (mg/dL) by treatment: olanzapine oral vs. placebo**	40.71	32.74	48.67	Norm	40.71	4.06	Pillinger 2020[106]
**Mean difference of triglycerides (mg/dL) by treatment: quetiapine oral vs. placebo**	28.32	18.58	38.94	Norm	28.32	5.19	Pillinger 2020[106]
**Mean difference of triglycerides (mg/dL) by treatment: risperidone oral vs. placebo**	3.54	-3.54	10.62	Norm	3.54	3.61	Pillinger 2020[106]
**Mean difference of triglycerides (mg/dL) by treatment: clozapine oral vs. placebo**	86.73	42.48	131.86	Norm	86.73	22.80	Pillinger 2020[106]
**Mean difference of triglycerides (mg/dL) by treatment: aripiprazole LAI vs. placebo**	1.77	-6.19	9.73	Norm	1.77	4.06	Pillinger 2020[106]
**Mean difference of triglycerides (mg/dL) by treatment: paliperidone LAI vs. placebo**	3.54	-3.54	10.62	Norm	3.54	3.61	Pillinger 2020[106]
**Mean difference of triglycerides (mg/dL) by treatment: risperidone LAI vs. placebo**	3.54	-3.54	10.62	Norm	3.54	3.61	Pillinger 2020[106]
**Mean difference of fasting glucose (mg/dL) by treatment: amisulpride oral vs. placebo**	-8.29	-24.86	8.47	Norm	-8.29	8.50	Pillinger 2020[106]
**Mean difference of fasting glucose (mg/dL) by treatment: aripiprazole oral vs. placebo**	2.34	-0.90	5.59	Norm	2.34	1.65	Pillinger 2020[106]
**Mean difference of fasting glucose (mg/dL) by treatment: cariprazine oral vs. placebo**	4.68	-0.90	10.45	Norm	4.68	2.90	Pillinger 2020[106]
**Mean difference of fasting glucose (mg/dL) by treatment: lurasidone oral vs. placebo**	-5.23	-9.91	-0.54	Norm	-5.23	2.39	Pillinger 2020[106]
**Mean difference of fasting glucose (mg/dL) by treatment: olanzapine oral vs. placebo**	3.60	-0.72	6.67	Norm	3.60	1.88	Pillinger 2020[106]
**Mean difference of fasting glucose (mg/dL) by treatment: quetiapine oral vs. placebo**	1.62	-1.98	5.23	Norm	1.62	1.84	Pillinger 2020[106]
**Mean difference of fasting glucose (mg/dL) by treatment: risperidone oral vs. placebo**	1.44	-1.08	3.96	Norm	1.44	1.29	Pillinger 2020[106]

Model parameter	Base-case	DSA parameter		PSA parameter			Source
		Low	High	Distribution	Alpha	Beta	
**Mean difference of fasting glucose (mg/dL) by treatment: clozapine oral vs. placebo**	18.92	7.39	30.63	Norm	18.92	5.93	Pillinger 2020[106]
**Mean difference of fasting glucose (mg/dL) by treatment: aripiprazole LAI vs. placebo**	2.34	-0.90	5.59	Norm	2.34	1.65	Pillinger 2020[106]
**Mean difference of fasting glucose (mg/dL) by treatment: paliperidone LAI vs. placebo**	1.44	-1.08	3.96	Norm	1.44	1.29	Pillinger 2020[106]
**Mean difference of fasting glucose (mg/dL) by treatment: risperidone LAI vs. placebo**	1.44	-1.08	3.96	Norm	1.44	1.29	Pillinger 2020[106]
**Mean difference of SBP (mmHg) by treatment: amisulpride oral vs. placebo**	1.17	-3.03	5.37	Norm	1.17	2.14	Gupta 2014[108]
**Mean difference of SBP (mmHg) by treatment: aripiprazole oral vs. placebo**	0.84	-3.32	5.00	Norm	0.84	2.12	Gupta 2014[108]
**Mean difference of SBP (mmHg) by treatment: cariprazine oral vs. placebo**	4.17	0.40	7.94	Norm	4.17	1.93	Greger 2021[107]
**Mean difference of SBP (mmHg) by treatment: lurasidone oral vs. placebo**	-1.59	-2.77	-0.41	Norm	-1.59	0.60	Greger 2021[107]
**Mean difference of SBP (mmHg) by treatment: olanzapine oral vs. placebo**	1.28	-0.15	2.71	Norm	1.28	0.73	Greger 2021[107]
**Mean difference of SBP (mmHg) by treatment: quetiapine oral vs. placebo**	2.60	0.00	5.20	Norm	2.60	1.33	Gupta 2014[108]
**Mean difference of SBP (mmHg) by treatment: risperidone oral vs. placebo**	2.60	0.00	5.20	Norm	2.60	1.33	Gupta 2014[108]
**Mean difference of SBP (mmHg) by treatment: clozapine oral vs. placebo**	3.90	-2.59	10.39	Norm	3.90	3.31	Gupta 2014[108]
**Mean difference of SBP (mmHg) by treatment: aripiprazole LAI vs. placebo**	0.84	-3.32	5.00	Norm	0.84	2.12	Gupta 2014[108]
**Mean difference of SBP (mmHg) by treatment: paliperidone LAI vs. placebo**	2.60	0.00	5.20	Norm	2.60	1.33	Assume = Risperidone
**Mean difference of SBP (mmHg) by treatment: risperidone LAI vs. placebo**	2.60	0.00	5.20	Norm	2.60	1.33	Gupta 2014[108]
**Intercept of logistic regression of diabetes**	-5.52	0.00	0.00	NA	0.00	0.00	Wilson 2007[109]
**Odds ratio of diabetes by age (50-64 vs. <50, year)**	0.98	0.64	1.50	Lognorm	-0.02	0.22	Wilson 2007[109]
**Odds ratio of diabetes by age (>=65 vs. <50, year)**	0.92	0.54	1.59	Lognorm	-0.08	0.28	Wilson 2007[109]
**Odds ratio of diabetes by male**	0.99	0.70	1.41	Lognorm	-0.01	0.18	Wilson 2007[109]
**Odds ratio of diabetes by parental diabetes (yes vs. no)**	1.76	1.17	2.64	Lognorm	0.57	0.21	Wilson 2007[109]
**Odds ratio of diabetes by BMI (25-30 vs. <25, kg/m2)**	1.35	0.78	2.34	Lognorm	0.30	0.28	Wilson 2007[109]
**Odds ratio of diabetes by BMI (?30 vs. <25, kg/m2)**	2.50	1.45	4.30	Lognorm	0.92	0.28	Wilson 2007[109]
**Odds ratio of diabetes by SBP > 130/85 mmHg OR receiving therapy**	1.65	1.10	2.46	Lognorm	0.50	0.21	Wilson 2007[109]
**Odds ratio of diabetes by HDL (men: < 40 mg/dL; women: <50 mg/dL)**	2.57	1.75	3.77	Lognorm	0.94	0.20	Wilson 2007[109]
**Odds ratio of diabetes by triglyceride level >= 150 mg/dL**	1.78	1.22	2.59	Lognorm	0.58	0.19	Wilson 2007[109]
**Odds ratio of diabetes by fasting glucose level >= 100 mg/dL**	7.25	4.89	10.74	Lognorm	1.98	0.20	Wilson 2007[109]
**Intercept of accelerated failure time weibull model of incident CHD in male**	12.79	6.40	19.18	Norm	12.79	3.26	D'Agostino 2000[110]
**Accelerated ratio of incident CHD in male by age (year)**	0.96	0.94	0.98	Lognorm	-0.04	0.01	D'Agostino 2000[110]
**Accelerated ratio of incident CHD in male by ln (total cholesterol/HDL)**	0.39	0.24	0.62	Lognorm	-0.95	0.24	D'Agostino 2000[110]
**Accelerated ratio of incident CHD in male by ln (SBP) (lnmmHg)**	0.36	0.20	0.66	Lognorm	-1.02	0.31	D'Agostino 2000[110]
**Accelerated ratio of incident CHD in male by If antihypertensive, (200 - SBP) * (SBP - 110) / 100 (mmHg)**	0.98	0.97	1.00	Lognorm	-0.02	0.01	D'Agostino 2000[110]
**Accelerated ratio of incident CHD in male by diabetes**	0.64	0.39	1.05	Lognorm	-0.44	0.25	D'Agostino 2000[110]
**Accelerated ratio of incident CHD in male by current smoker**	0.55	0.40	0.74	Lognorm	-0.60	0.15	D'Agostino 2000[110]
**Scale of accelerated failure time weibull model of incident CHD in male**	0.78	0.00	0.00	NA	0.00	0.00	D'Agostino 2000[110]
**Intercept of accelerated failure time weibull model of incident CHD in female**	20.97	10.49	31.45	Norm	20.97	5.35	D'Agostino 2000[110]
**Accelerated ratio of incident CHD in female by age (year)**	0.94	0.89	1.00	Lognorm	-0.06	0.03	D'Agostino 2000[110]
**Accelerated ratio of incident CHD in female by menopause**	0.02	0.00	0.40	Lognorm	-3.85	1.50	D'Agostino 2000[110]
**Accelerated ratio of incident CHD in female by age AND menopause (year)**	1.08	1.01	1.14	Lognorm	0.07	0.03	D'Agostino 2000[110]
**Accelerated ratio of incident CHD in female by ln (total cholesterol/HDL)**	0.53	0.32	0.91	Lognorm	-0.63	0.27	D'Agostino 2000[110]
**Accelerated ratio of incident CHD in female by ln (SBP) (lnmmHg)**	0.11	0.03	0.33	Lognorm	-2.24	0.57	D'Agostino 2000[110]
**Accelerated ratio of incident CHD in female by (200 - SBP) * (SBP - 110) / 100 (mmHg) AND using antihypertensive therapy**	0.91	0.75	1.09	Lognorm	-0.10	0.09	D'Agostino 2000[110]
**Accelerated ratio of incident CHD in female by diabetes**	0.59	0.37	0.95	Lognorm	-0.52	0.24	D'Agostino 2000[110]
**Accelerated ratio of incident CHD in female by current smoker**	0.69	0.50	0.94	Lognorm	-0.38	0.16	D'Agostino 2000[110]

Model parameter	Base-case	DSA parameter		PSA parameter			Source
		Low	High	Distribution	Alpha	Beta	
**Accelerated ratio of incident CHD in female by ln (triglycerides) (ln mg/dL)**	1.31	1.01	1.69	Lognorm	0.27	0.13	D'Agostino 2000[110]
**Accelerated ratio of incident CHD in female by alcohol (ow/wk)**	1.05	1.00	1.12	Lognorm	0.05	0.03	D'Agostino 2000[110]
**Scale of accelerated failure time weibull model of incident CHD in female**	0.75	0.00	0.00	NA	0.00	0.00	D'Agostino 2000[110]
**Intercept of accelerated failure time weibull model of subsequent CHD in male**	5.00	2.50	7.49	Norm	5.00	1.27	D'Agostino 2000[110]
**Accelerated ratio of subsequent CHD in male by age (year)**	0.99	0.97	1.00	Lognorm	-0.01	0.01	D'Agostino 2000[110]
**Accelerated ratio of subsequent CHD in male by ln (total cholesterol/HDL)**	0.51	0.33	0.78	Lognorm	-0.67	0.22	D'Agostino 2000[110]
**Accelerated ratio of subsequent CHD in male by diabetes**	0.74	0.52	1.04	Lognorm	-0.30	0.17	D'Agostino 2000[110]
**Scale of accelerated failure time weibull model of subsequent CHD in male**	1.00	0.00	0.00	NA	0.00	0.00	D'Agostino 2000[110]
**Intercept of accelerated failure time weibull model of subsequent CHD in female**	13.54	5.50	21.57	Norm	13.54	4.10	D'Agostino 2000[110]
**Accelerated ratio of subsequent CHD in female by age (year)**	0.98	0.94	1.01	Lognorm	-0.02	0.02	D'Agostino 2000[110]
**Accelerated ratio of subsequent CHD in female by ln (total cholesterol/HDL)**	0.43	0.20	0.92	Lognorm	-0.83	0.38	D'Agostino 2000[110]
**Accelerated ratio of subsequent CHD in female by ln (SBP) (lnmmHg)**	0.25	0.05	1.30	Lognorm	-1.37	0.83	D'Agostino 2000[110]
**Accelerated ratio of subsequent CHD in female by diabetes**	0.46	0.26	0.80	Lognorm	-0.78	0.28	D'Agostino 2000[110]
**Accelerated ratio of subsequent CHD in female by current smoker**	0.69	0.40	1.19	Lognorm	-0.37	0.27	D'Agostino 2000[110]
**Scale of accelerated failure time weibull model of subsequent CHD in female**	1.03	0.00	0.00	NA	0.00	0.00	D'Agostino 2000[110]
**Rate of exponential model of stroke in male**	0.00	0.00	0.00	NA	0.00	0.00	D'Agostino 1994[111]
**Hazard ratio of stroke in male by age (centre at 50 per 10 years)**	1.05	1.03	1.07	Lognorm	0.05	0.01	D'Agostino 1994[111]
**Hazard ratio of stroke in male by SBP (centred at 110 per 10 mmHg)**	1.02	1.01	1.02	Lognorm	0.02	0.00	D'Agostino 1994[111]
**Hazard ratio of stroke in male by (200 - SBP) * (SBP - 110) / 100 (mmHg) AND using antihypertensive therapy**	1.00	1.00	1.00	Lognorm	0.00	0.00	D'Agostino 1994[111]
**Hazard ratio of stroke in male by CVD**	1.73	1.68	1.78	Lognorm	0.55	0.02	D'Agostino 1994[111]
**Hazard ratio of stroke in male by left ventricular hypertrophy**	2.20	1.26	3.84	Lognorm	0.79	0.28	D'Agostino 1994[111]
**Hazard ratio of stroke in male by Current smoker**	1.69	1.27	2.23	Lognorm	0.52	0.14	D'Agostino 1994[111]
**Hazard ratio of stroke in male by atrial fabriation**	1.82	1.01	3.29	Lognorm	0.60	0.30	D'Agostino 1994[111]
**Hazard ratio of stroke in male by diabetes**	1.41	0.97	2.04	Lognorm	0.34	0.19	D'Agostino 1994[111]
**Rate of exponential model of stroke in female**	0.00	0.00	0.00	NA	0.00	0.00	D'Agostino 1994[111]
**Hazard ratio of stroke in female by age (centre at 50 per 10 years)**	1.07	1.05	1.09	Lognorm	0.07	0.01	D'Agostino 1994[111]
**Hazard ratio of stroke in female by SBP (centred at 110 per 10 mmHg)**	1.02	1.01	1.02	Lognorm	0.02	0.00	D'Agostino 1994[111]
**Hazard ratio of stroke in female by (200 - SBP) * (SBP - 110) / 100 (mmHg) AND using antihypertensive therapy**	1.00	1.00	1.00	Lognorm	0.00	0.00	D'Agostino 1994[111]
**Hazard ratio of stroke in female by CVD**	1.55	1.17	2.07	Lognorm	0.44	0.15	D'Agostino 1994[111]
**Hazard ratio of stroke in female by left ventricular hypertrophy**	2.24	1.39	3.60	Lognorm	0.81	0.24	D'Agostino 1994[111]
**Hazard ratio of stroke in female by current smoker**	1.72	1.29	2.29	Lognorm	0.54	0.15	D'Agostino 1994[111]
**Hazard ratio of stroke in female by atrial fabriation**	3.06	1.95	4.80	Lognorm	1.12	0.23	D'Agostino 1994[111]
**Hazard ratio of stroke in female by diabetes**	1.75	1.25	2.45	Lognorm	0.56	0.17	D'Agostino 1994[111]
**SMR of death in schizophrenia in male**	4.10	2.80	5.90	Lognorm	1.41	0.19	Reininghaus 2014[112]
**SMR of death in schizophrenia in female**	2.80	1.60	5.10	Lognorm	1.03	0.30	Reininghaus 2014[112]
**Proportion of death at stroke in male**	0.26	0.26	0.27	Beta	7882	21974	Seminog 2019[113]
**Proportion of death at stroke in female**	0.29	0.21	0.29	Beta	142.71	358.02	Seminog 2019[113]
**Proportion of death at CHD in male**	0.08	0.08	0.08	Beta	16823	198181	Asaria 2017[114]
**Proportion of death at CHD in female**	0.12	0.12	0.12	Beta	15872	113269	Asaria 2017[114]
**Proportion of death at agranulocytosis**	0.02	0.00	0.00	Beta	2.00	91.00	Munro 1999[104]
**Utility weight at stable state of schizophrenia**	0.81	0.74	0.88	Beta	97.72	23.37	Lenert 2004[115]
**Utility weight at relapse state of schizophrenia**	0.54	0.42	0.65	Beta	38.40	32.76	Lenert 2004[115]
**Utility weight by agranulocytosis**	0.46	0.20	0.90	Beta	3.12	3.67	Week 1991[116]

Model parameter	Base-case	DSA parameter		PSA parameter			Source
		Low	High	Distribution	Alpha	Beta	
**Utility decrement due to EPS**	-0.04	-0.06	-0.03	Norm	-0.04	0.01	Millier 2014[117]
**Utility decrement due to sedation**	-0.02	-0.03	0.00	Norm	-0.02	0.01	Millier 2014[117]
**Utility decrement due to sexual dysfunction**	-0.02	-0.04	0.00	Norm	-0.02	0.01	Millier 2014[117]
**Utility decrement due to weight gain**	-0.02	-0.04	-0.01	Norm	-0.02	0.01	Millier 2014[117]
**Utility decrement due to tardive dyskinesia**	-0.04	-0.06	-0.03	Norm	-0.04	0.01	Assume = EPS
**Utility decrement due to diabetes**	-0.15	-0.19	-0.11	Norm	-0.15	0.02	Briggs 2008[118]
**Utility decrement due to CHD**	-0.06	-0.07	-0.04	Norm	-0.06	0.01	Clarke 2002[119]
**Utility decrement due to stroke**	-0.16	-0.22	-0.11	Norm	-0.16	0.03	Clarke 2002[119]
**Annual drug tariff of amisulpride oral**	86	64	107	Gamma	61.47	1.40	BNF 79[120] [Range: ±25%]
**Annual drug tariff of aripiprazole oral**	24	18	30	Gamma	61.47	0.39	BNF 79[120] [Range: ±25%]
**Annual drug tariff of cariprazine oral**	1048	786	1310	Gamma	61.47	17.05	BNF 79[120] [Range: ±25%]
**Annual drug tariff of lurasidone oral**	1183	888	1479	Gamma	61.47	19.25	BNF 79[120] [Range: ±25%]
**Annual drug tariff of olanzapine oral**	23	17	29	Gamma	61.47	0.37	BNF 79[120] [Range: ±25%]
**Annual drug tariff of quetiapine oral**	93	70	117	Gamma	61.47	1.52	BNF 79[120] [Range: ±25%]
**Annual drug tariff of risperidone oral**	160	120	200	Gamma	61.47	2.60	BNF 79[120] [Range: ±25%]
**Annual drug tariff of clozapine oral**	868	651	1085	Gamma	61.47	14.12	BNF 79[120] [Range: ±25%]
**Annual drug tariff of aripiprazole LAI**	2645	1984	3306	Gamma	61.47	43.03	BNF 79[120] [Range: ±25%]
**Annual drug tariff of paliperidone LAI**	3772	2829	4715	Gamma	61.47	61.36	BNF 79[120] [Range: ±25%]
**Annual drug tariff of risperidone LAI**	1375	1031	1718	Gamma	61.47	22.36	BNF 79[120] [Range: ±25%]
**Cost per episode of agranulocytosis**	858	644	1073	Gamma	61.47	13.96	NHS reference 2020[121] [Range: ±25%]
**Cost per episode of EPS**	225	168	281	Gamma	61.47	3.65	NICE 2014[96], NHS reference 2020[121], BNF79[120] [Range: ±25%]
**Cost per episode of sedation**	39	29	49	Gamma	61.47	0.63	Assumption, PSSRU 2020[122] [Range: ±25%]
**Cost per episode of sexual dysfunction**	39	29	49	Gamma	61.47	0.63	
**Cost per episode of weight gain**	98	74	123	Gamma	61.47	1.59	
**Cost per episode of tardive dyskinesia**	225	168	281	Gamma	61.47	3.65	Assume = EPS
**Annual cost of CHD in people without prior CVD in year of CHD**	4530	4232	4828	Norm	4530	152	Zhou 2023[123]
**Annual cost of CHD in people without prior CVD in 1 year after CHD**	560	464	656	Norm	560	49	Zhou 2023[123]
**Annual cost of CHD in people without prior CVD in 2 years after CHD**	380	297	463	Norm	380	42	Zhou 2023[123]
**Annual cost of CHD in people without prior CVD in ≥3 years after CHD**	350	283	417	Norm	350	34	Zhou 2023[123]
**Annual cost of CHD in people with prior CVD in year of CHD**	5840	5393	6287	Norm	5840	228	Zhou 2023[123]
**Annual cost of CHD in people with prior CVD in 1 year after CHD**	940	664	1216	Norm	940	141	Zhou 2023[123]
**Annual cost of CHD in people with prior CVD in 2 years after CHD**	600	433	767	Norm	600	85	Zhou 2023[123]
**Annual cost of CHD in people with prior CVD in ≥3 years after CHD**	600	433	767	Norm	600	85	Zhou 2023[123]
**Annual cost of stroke in people without prior CVD in year of stroke**	5950	5677	6223	Norm	5950	139	Zhou 2023[123]
**Annual cost of stroke in people without prior CVD in 1 year after stroke**	1420	1205	1635	Norm	1420	110	Zhou 2023[123]
**Annual cost of stroke in people without prior CVD in 2 years after stroke**	770	631	909	Norm	770	71	Zhou 2023[123]
**Annual cost of stroke in people without prior CVD in ≥3 years after stroke**	670	537	803	Norm	670	68	Zhou 2023[123]
**Annual cost of stroke in people with prior CVD in year of stroke**	6600	6178	7022	Norm	6600	215	Zhou 2023[123]
**Annual cost of stroke in people with prior CVD in 1 year after stroke**	1610	1317	1903	Norm	1610	149	Zhou 2023[123]
**Annual cost of stroke in people with prior CVD in 2 years after stroke**	1240	984	1496	Norm	1240	131	Zhou 2023[123]
**Annual cost of stroke in people with prior CVD in ≥3 years after stroke**	1240	984	1496	Norm	1240	131	Zhou 2023[123]
**Annual cost of diabetes in people without prior CVD in < 10 years after diabetes**	520	495	545	Norm	520	13	Zhou 2023[123]
**Annual cost of diabetes in people without prior CVD in ≥10 years after diabetes**	650	603	697	Norm	650	24	Zhou 2023[123]
**Annual cost of diabetes in people with prior CVD in < 10 years after diabetes**	650	579	721	Norm	650	36	Zhou 2023[123]

Model parameter	Base-case	DSA parameter		PSA parameter			Source
		Low	High	Distribution	Alpha	Beta	
**Annual cost of diabetes in people with prior CVD in ≥10 years after diabetes**	810	713	907	Norm	810	50	Zhou 2023[123]
**Cost of death due to CHD or stroke in people without prior CVD**	1460	1135	1785	Norm	1460	166	Zhou 2023[123]
**Cost of death due to CHD or stroke in people with prior CVD**	2700	2226	3174	Norm	2700	242	Zhou 2023[123]
**Cost of death due to the other reasons in people without prior CVD**	5740	5514	5966	Norm	5740	115	Zhou 2023[123]
**Cost of death due to the other reasons in people with prior CVD**	6240	5805	6675	Norm	6240	222	Zhou 2023[123]
**Annual cost of schizophrenia state of stable**	6648	3324	9971	Gamma	15	433	NICE 2014[96], PSSRU 2020[122] [Range: ±50%]
**Annual cost of schizophrenia state of relapse at non-acute phase**	5321	2660	7981	Gamma	15	346	
**Cost of managing acute phase of a relapse episode**	28645	14323	42968	Gamma	15	1864	

BMI, Body mass index; CHD, coronary heart disease; CVD, cardiovascular disease; DSA, deterministic sensitivity analysis; EPS, extrapyramidal symptom; HDL, high density lipoprotein; PSA, probabilistic sensitivity analysis; SBP, systolic blood pressure; SMR, standardized mortality ratio.

Supplementary table 3. Base-case incremental outcomes over 10 years under compared first-line antipsychotics

Reference	Ranking at WTP £20K/QALY	Incremental costs (£) / Incremental QALYs [ICER, £/QALY] compared to the reference								
		**AMI**	**RIS-LAI**	**ARI-LAI**	**OLA**	**PAL-LAI**	**RIS**	**ARI**	**LUR**	**QUE**
**AMI**	1	..	..	..	..	..	..	..	..	..
**RIS-LAI**	3	-6500 / 0.038	..	..	..	..	..	..	..	..
**ARI-LAI**	5	-13500 / 0.052	-7000 / 0.014	..	..	..	..	..	..	..
**OLA**	2	-1900 / 0.064	**4600 / 0.026 [179300]**	**11600 / 0.012 [955300]**	..	..	..	..	..	..
**PAL-LAI**	7	-17800 / 0.070	-11300 / 0.032	-4300 / 0.019	-15900 / 0.006	..	..	..	..	..
**RIS**	4	-10200 / 0.074	-3700 / 0.037	**3300 / 0.023 [144400]**	-8300 / 0.011	**7600 / 0.004 [1818000]**	..	..	..	..
**ARI**	6	-15600 / 0.095	-9100 / 0.057	-2100 / 0.044	-13700 / 0.031	**2200 / 0.025 [87700]**	-5400 / 0.021	..	..	..
**LUR**	8	-26500 / 0.125	-20000 / 0.087	-13000 / 0.073	-24600 / 0.061	-8700 / 0.054	-16300 / 0.050	-10900 / 0.029	..	..
**QUE**	9	-20300 / 0.132	-13700 / 0.094	-6800 / 0.080	-18400 / 0.068	-2500 / 0.061	-10100 / 0.057	-4700 / 0.036	**6200 / 0.007 [898700]**	..
**CAR**	10	-27200 / 0.174	-20700 / 0.137	-13700 / 0.123	-25400 / 0.111	-9500 / 0.104	-17000 / 0.100	-11600 / 0.079	-700 / 0.050	-7000 / 0.043

Both costs and QALY were discounted; Costs, incremental costs and ICER were round to 100; QALYs and incremental QALYs were round to 0.001.
References and comparators sorted by decreasing QALYs (thus incremental QALYs were all positive). ICER was not estimated if incremental costs was negative (reference was dominated).
Treatment is an oral one if its name is not followed by "LAI"; AMI, amisulpride; ARI, aripiprazole; CAR, cariprazine; ICER, incremental cost-effectiveness ratio; LAI, long-acting injection; LUR, lurasidone; OLA, olanzapine; PAL, paliperidone; QALY, quality-adjusted life year; QUE, quetiapine; RIS, risperidone; WTP. Willingness to pay

Supplementary table 4. Cost-effectiveness results across all the scenarios.

Scenario		Outcome		Incremental outcome		ICER (£/QALY gained)
1st line	2nd line	Cost (£)	QALY	Cost (£)	QALY	
**Undominated scenarios**						
**AMI**	**OLA**	138,218	6.153	-	-	-
**AMI**	**RIS-LAI**	141,318	6.156	3,100	0.003	909,148
**All scenarios**						
**AMI**	**OLA**	138,218	6.153	-	-	-
**OLA**	**AMI**	139,101	6.099	883	-0.054	Dominated
**AMI**	**RIS-LAI**	141,318	6.156	3,100	0.003	909,148
**OLA**	**RIS-LAI**	143,192	6.091	1,873	-0.066	Dominated
**AMI**	**RIS**	143,306	6.133	1,987	-0.023	Dominated
**RIS-LAI**	**AMI**	143,858	6.127	2,540	-0.029	Dominated
**RIS-LAI**	**OLA**	144,791	6.115	3,473	-0.041	Dominated
**OLA**	**RIS**	145,217	6.067	3,899	-0.089	Dominated
**AMI**	**ARI-LAI**	146,172	6.146	4,854	-0.010	Dominated
**AMI**	**ARI**	146,507	6.118	5,189	-0.039	Dominated
**RIS**	**AMI**	147,067	6.091	5,748	-0.065	Dominated
**RIS**	**OLA**	148,077	6.077	6,759	-0.079	Dominated
**OLA**	**ARI-LAI**	148,160	6.081	6,842	-0.076	Dominated
**OLA**	**ARI**	148,484	6.053	7,165	-0.103	Dominated
**AMI**	**PAL-LAI**	149,387	6.136	8,068	-0.020	Dominated
**AMI**	**QUE**	149,811	6.098	8,493	-0.059	Dominated
**RIS-LAI**	**RIS**	150,021	6.095	8,703	-0.062	Dominated
**ARI-LAI**	**AMI**	150,940	6.115	9,621	-0.041	Dominated
**OLA**	**PAL-LAI**	151,425	6.071	10,107	-0.085	Dominated
**RIS**	**RIS-LAI**	151,622	6.082	10,304	-0.075	Dominated
**OLA**	**QUE**	151,855	6.033	10,537	-0.123	Dominated
**ARI-LAI**	**OLA**	151,914	6.102	10,595	-0.055	Dominated
**ARI**	**AMI**	152,249	6.072	10,930	-0.084	Dominated
**RIS-LAI**	**ARI-LAI**	153,349	6.107	12,031	-0.049	Dominated
**ARI**	**OLA**	153,352	6.057	12,034	-0.099	Dominated
**RIS-LAI**	**ARI**	153,438	6.079	12,119	-0.077	Dominated
**AMI**	**LUR**	154,843	6.084	13,524	-0.072	Dominated
**AMI**	**CAR**	155,213	6.069	13,894	-0.088	Dominated
**PAL-LAI**	**AMI**	155,395	6.097	14,077	-0.060	Dominated
**ARI-LAI**	**RIS-LAI**	155,598	6.104	14,280	-0.052	Dominated
**PAL-LAI**	**OLA**	156,392	6.083	15,074	-0.073	Dominated
**RIS-LAI**	**PAL-LAI**	156,667	6.097	15,349	-0.059	Dominated
**RIS-LAI**	**QUE**	156,841	6.059	15,523	-0.097	Dominated
**QUE**	**AMI**	156,926	6.034	15,608	-0.122	Dominated
**OLA**	**LUR**	156,999	6.019	15,680	-0.138	Dominated
**RIS**	**ARI-LAI**	157,105	6.071	15,787	-0.086	Dominated
**ARI**	**RIS-LAI**	157,189	6.061	15,870	-0.095	Dominated
**RIS**	**ARI**	157,371	6.039	16,053	-0.117	Dominated
**OLA**	**CAR**	157,391	6.006	16,073	-0.151	Dominated
**ARI-LAI**	**RIS**	157,442	6.080	16,124	-0.076	Dominated
**QUE**	**OLA**	158,046	6.018	16,728	-0.138	Dominated
**ARI**	**RIS**	159,356	6.033	18,038	-0.123	Dominated
**PAL-LAI**	**RIS-LAI**	160,167	6.086	18,849	-0.071	Dominated
**RIS**	**PAL-LAI**	160,680	6.060	19,361	-0.097	Dominated
**ARI-LAI**	**ARI**	160,981	6.064	19,662	-0.092	Dominated
**RIS**	**QUE**	161,057	6.017	19,738	-0.139	Dominated
**PAL-LAI**	**RIS**	162,047	6.060	20,729	-0.096	Dominated
**QUE**	**RIS-LAI**	162,078	6.023	20,760	-0.133	Dominated
**RIS-LAI**	**LUR**	162,097	6.043	20,779	-0.113	Dominated
**RIS-LAI**	**CAR**	162,508	6.029	21,190	-0.128	Dominated
**ARI**	**ARI-LAI**	163,024	6.049	21,705	-0.107	Dominated
**LUR**	**AMI**	163,318	6.046	22,000	-0.110	Dominated
**CAR**	**AMI**	164,035	5.990	22,716	-0.167	Dominated
**QUE**	**RIS**	164,352	5.995	23,034	-0.162	Dominated
**ARI-LAI**	**PAL-LAI**	164,387	6.083	23,069	-0.074	Dominated
**LUR**	**OLA**	164,536	6.026	23,217	-0.130	Dominated
**ARI-LAI**	**QUE**	164,545	6.042	23,226	-0.114	Dominated
**CAR**	**OLA**	165,251	5.972	23,932	-0.184	Dominated
**PAL-LAI**	**ARI-LAI**	165,641	6.074	24,322	-0.082	Dominated
**PAL-LAI**	**ARI**	165,676	6.044	24,358	-0.113	Dominated
**RIS**	**LUR**	166,596	6.001	25,277	-0.155	Dominated
**ARI**	**PAL-LAI**	166,800	6.037	25,481	-0.119	Dominated
**RIS**	**CAR**	167,018	5.985	25,700	-0.171	Dominated
**ARI**	**QUE**	167,044	5.992	25,726	-0.164	Dominated
**QUE**	**ARI-LAI**	168,197	6.011	26,878	-0.145	Dominated
**QUE**	**ARI**	168,369	5.977	27,050	-0.180	Dominated

Scenario		Outcome		Incremental outcome		ICER
1st line	2nd line	Cost (£)	QALY	Cost (£)	QALY	(£/QALY gained)
**LUR**	**RIS-LAI**	168,845	6.031	27,526	-0.126	Dominated
**PAL-LAI**	**QUE**	169,323	6.022	28,004	-0.134	Dominated
**CAR**	**RIS-LAI**	169,629	5.977	28,310	-0.179	Dominated
**ARI-LAI**	**LUR**	170,018	6.027	28,700	-0.130	Dominated
**ARI-LAI**	**CAR**	170,425	6.011	29,107	-0.145	Dominated
**LUR**	**RIS**	171,121	6.000	29,802	-0.156	Dominated
**CAR**	**RIS**	171,861	5.947	30,542	-0.209	Dominated
**QUE**	**PAL-LAI**	172,170	5.999	30,851	-0.158	Dominated
**ARI**	**LUR**	172,882	5.976	31,564	-0.180	Dominated
**ARI**	**CAR**	173,315	5.958	31,996	-0.198	Dominated
**PAL-LAI**	**LUR**	174,912	6.006	33,593	-0.151	Dominated
**LUR**	**ARI-LAI**	175,228	6.018	33,910	-0.139	Dominated
**PAL-LAI**	**CAR**	175,332	5.990	34,014	-0.167	Dominated
**LUR**	**ARI**	175,337	5.980	34,019	-0.177	Dominated
**CAR**	**ARI-LAI**	176,076	5.965	34,758	-0.192	Dominated
**CAR**	**ARI**	176,117	5.930	34,799	-0.227	Dominated
**QUE**	**LUR**	178,330	5.935	37,012	-0.221	Dominated
**QUE**	**CAR**	178,799	5.917	37,481	-0.240	Dominated
**LUR**	**PAL-LAI**	179,381	6.003	38,063	-0.153	Dominated
**LUR**	**QUE**	179,427	5.954	38,109	-0.203	Dominated
**CAR**	**QUE**	180,219	5.904	38,901	-0.253	Dominated
**CAR**	**PAL-LAI**	180,237	5.952	38,919	-0.205	Dominated
**LUR**	**CAR**	186,190	5.914	44,871	-0.242	Dominated
**CAR**	**LUR**	186,557	5.887	45,239	-0.270	Dominated

Treatment is an oral one if its name is not followed by “LAI”; AMI, amisulpride; ARI, aripiprazole; CAR, cariprazine; ICER, incremental cost effectiveness ratio; LAI, long-acting injection; LUR, lurasidone; OLA, olanzapine; PAL, paliperidone; QALY, quality-adjusted life year; QUE, quetiapine; RIS, risperidone;

Supplementary figures

Supplementary figure 1. Base-case rankings of cost-effectiveness among first-line antipsychotics

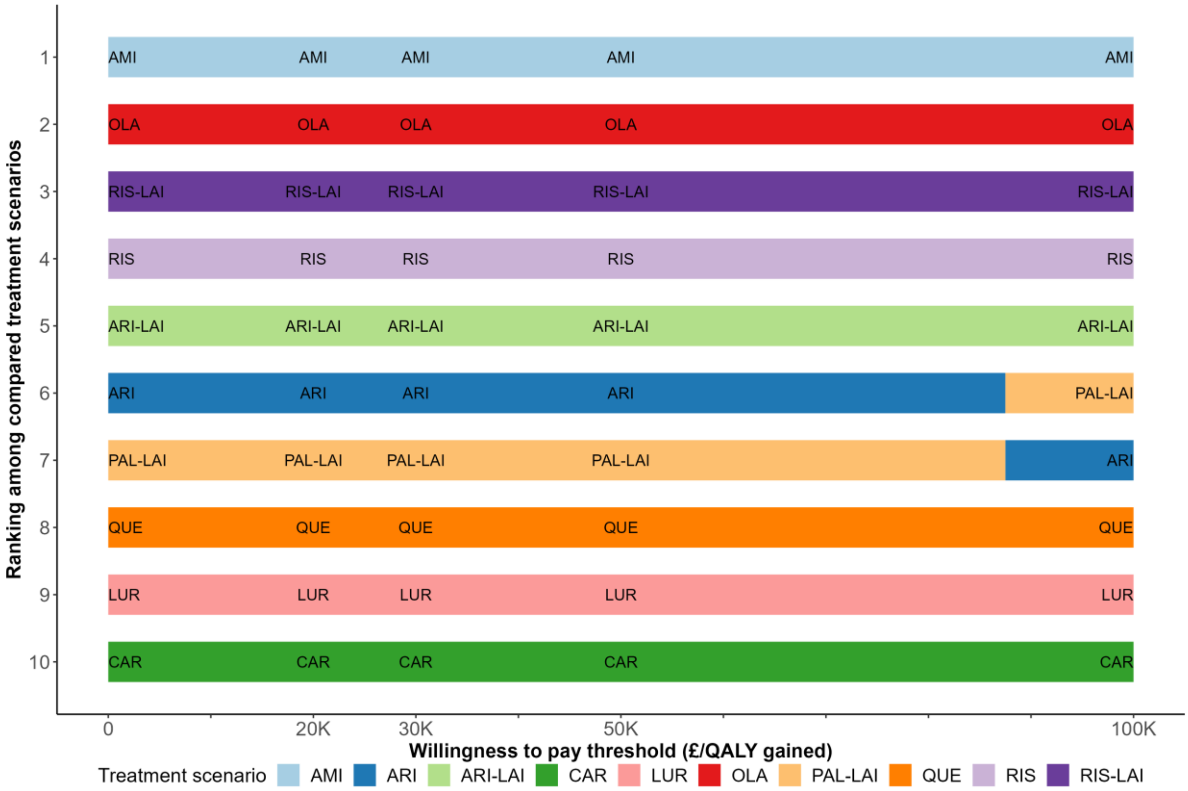

Treatment is an oral one if its name is not followed by "LAI"; AMI, amisulpride; ARI, aripiprazole; CAR, cariprazine; LAI, long-acting injection; LUR, lurasidone; OLA, olanzapine; PAL, paliperidone; QALY, quality-adjusted life year; QUE, quetiapine; RIS, risperidone;

Supplementary figure 2. Probabilistic sensitivity analyses results of the ranking among comparators

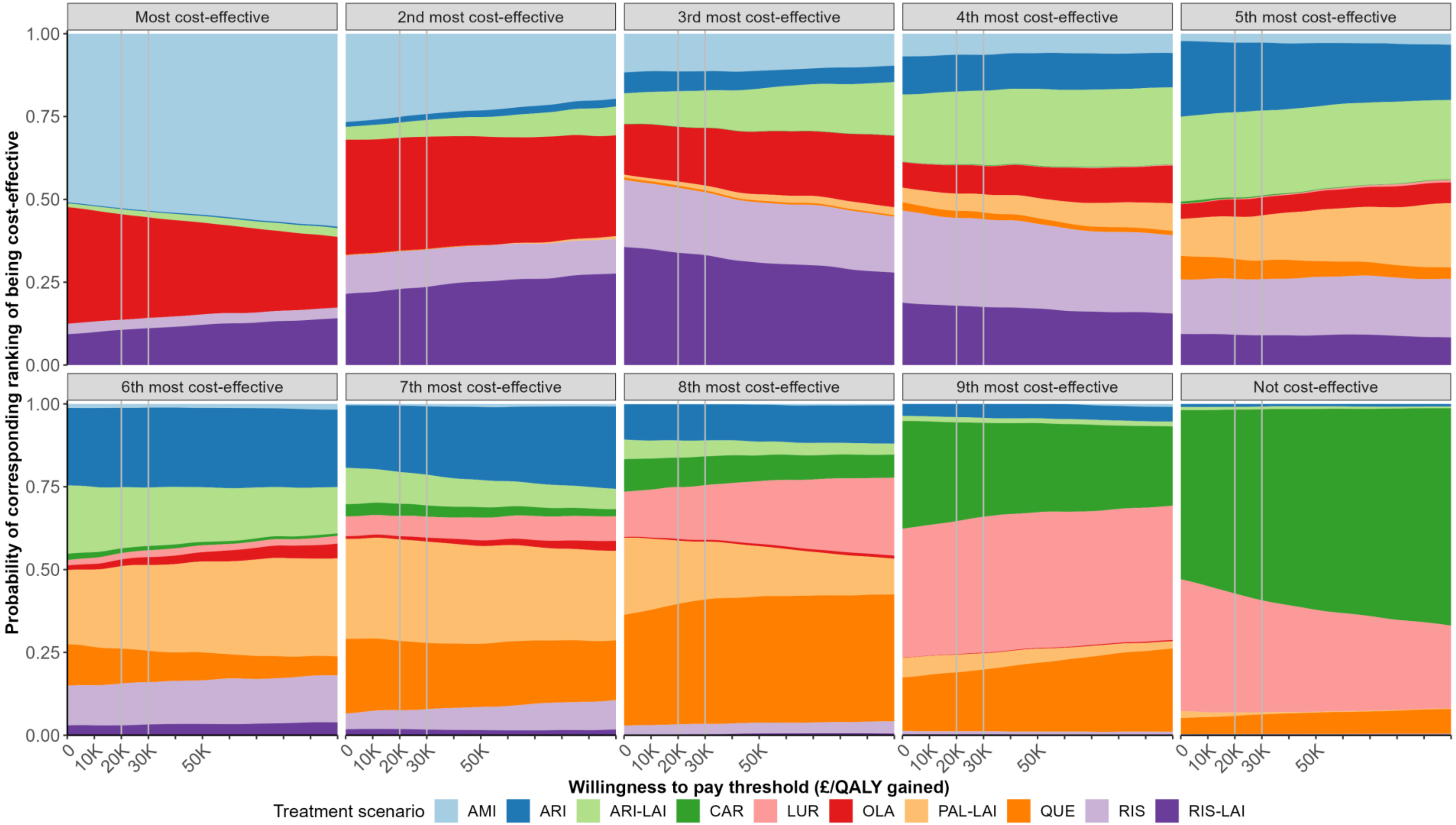

Treatment is an oral one if its name is not followed by "LAI"; AMI, amisulpride; ARI, aripiprazole; CAR, cariprazine; LAI, long-acting injection; LUR, lurasidone; OLA, olanzapine; PAL, paliperidone; QALY, quality-adjusted life year; QUE, quetiapine; RIS, risperidone;

Supplementary figure 3. Top 3 impactful inputs (n=263) on NMB at £20K/QALY in AMI vs. others

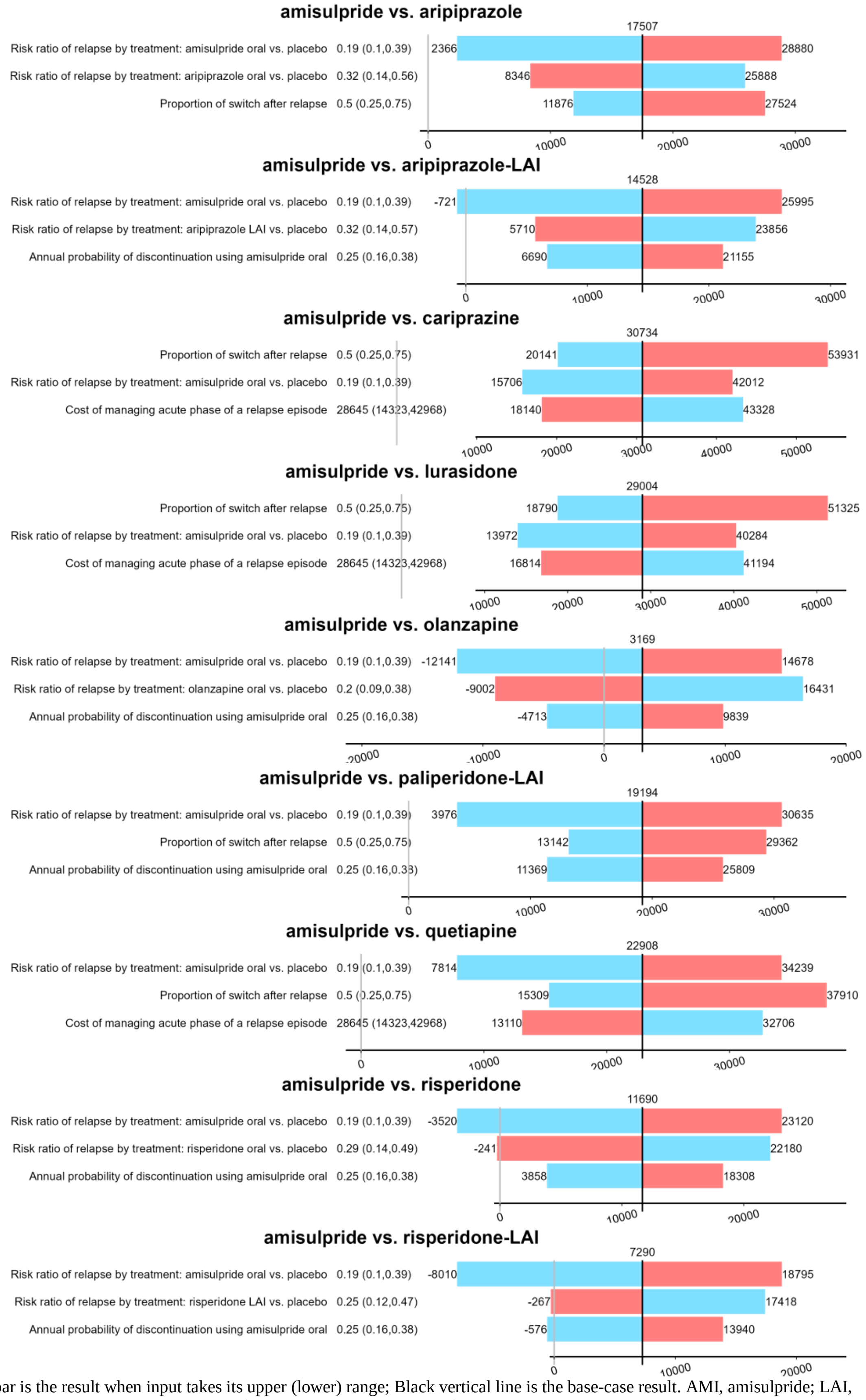

Blue (Red) bar is the result when input takes its upper (lower) range; Black vertical line is the base-case result. AMI, amisulpride; LAI, long-acting injection; NMB, net monetary benefit; QALY, quality-adjusted life year;
Comparison between other pairs of included antipsychotics is similar (results not shown)

Supplementary figure 4. Top 3 impactful inputs (n=263) on incremental QALYs in AMI vs. others

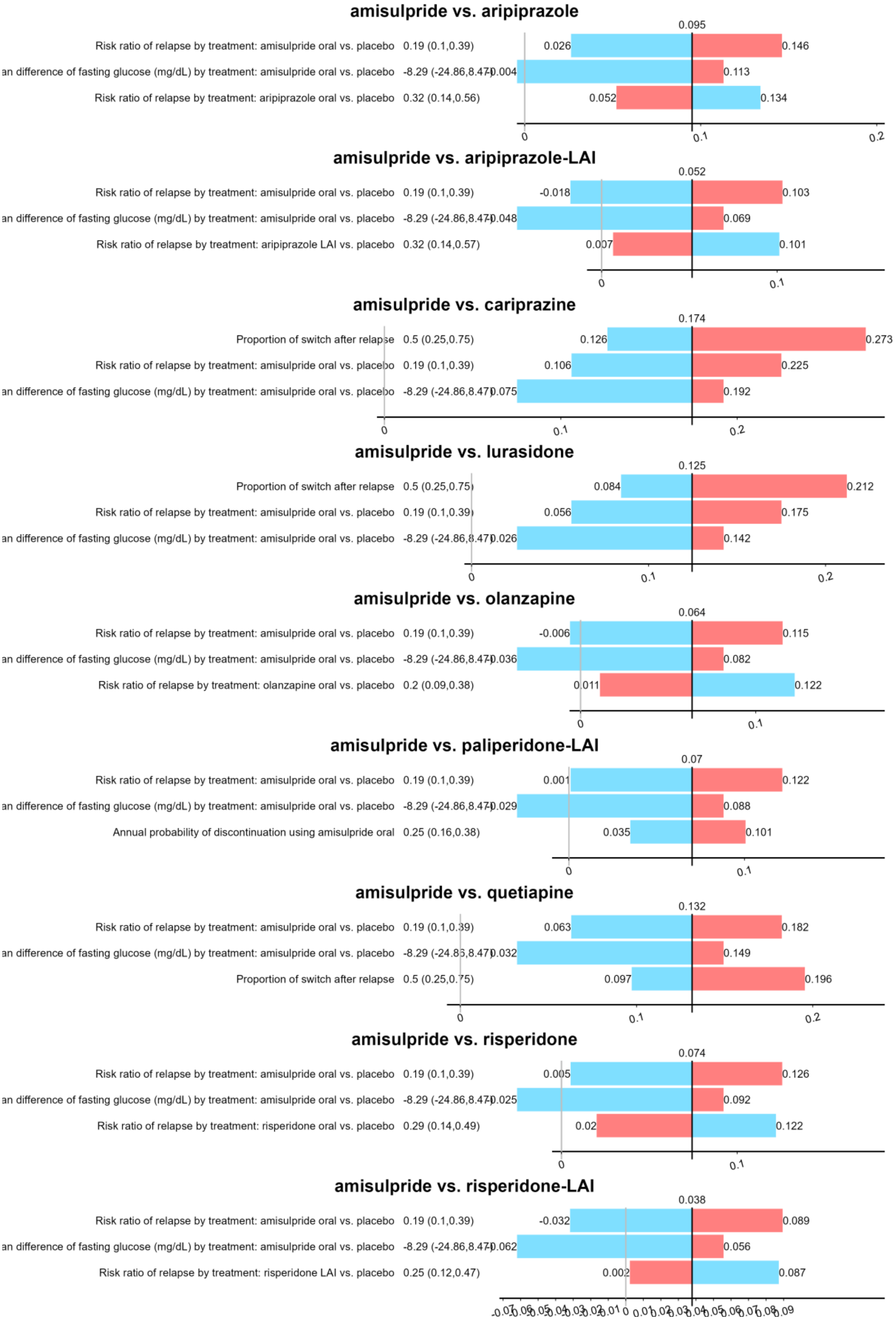

Blue (Red) bar is the result when input takes its upper (lower) range; Black vertical line is the base-case result. AMI, amisulpride; LAI, long-acting injection; QALY, quality-adjusted life year;
Comparison between other pairs of included antipsychotics is similar (results not shown)

Supplementary figure 5a. Scenario analyses results with different analytical timeframes

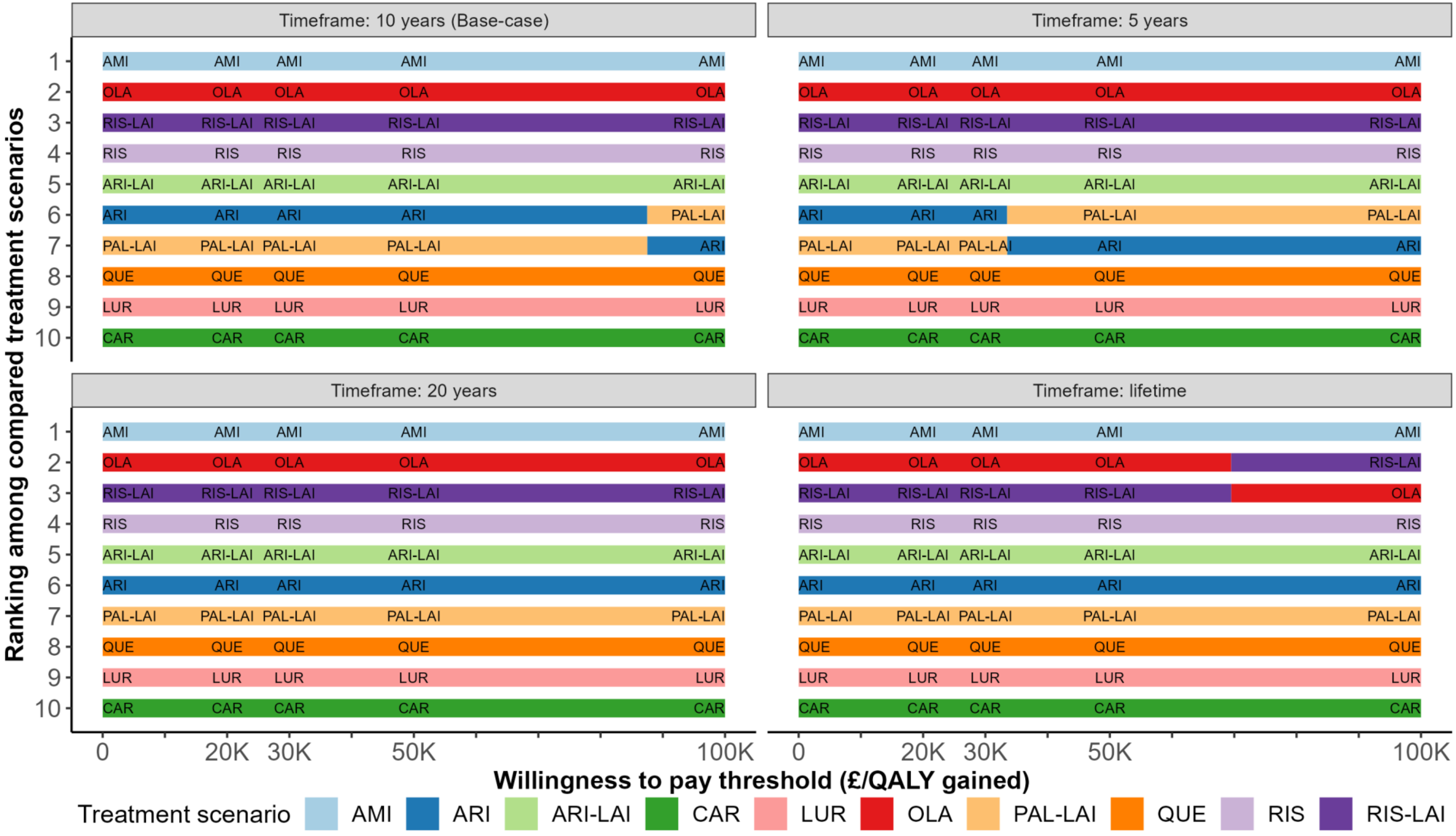

Treatment is an oral one if its name is not followed by "LAI"; AMI, amisulpride; ARI, aripiprazole; CAR, cariprazine; LAI, long-acting injection; LUR, lurasidone; OLA, olanzapine; PAL, paliperidone; QALY, quality-adjusted life year; QUE, quetiapine; RIS, risperidone;

Supplementary figure 5b. Scenario analyses results with different discount rates

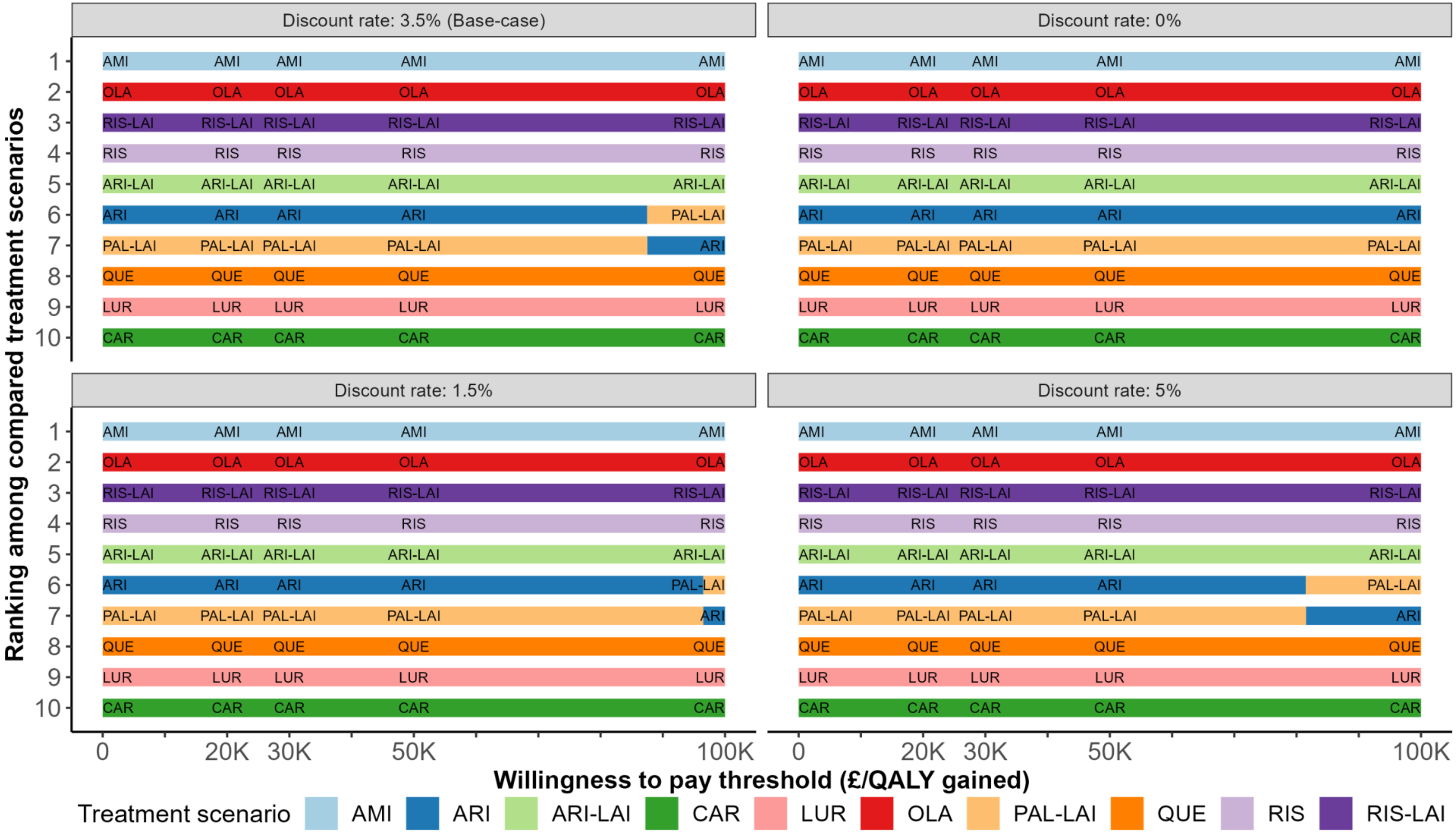

Treatment is an oral one if its name is not followed by “LAI”; AMI, amisulpride; ARI, aripiprazole; CAR, cariprazine; LAI, long-acting injection; LUR, lurasidone; OLA, olanzapine; PAL, paliperidone; QALY, quality-adjusted life year; QUE, quetiapine; RIS, risperidone;

Supplementary figure 6. Scenario analyses results comparing antipsychotics as second-line treatment in non-first-episode schizophrenia

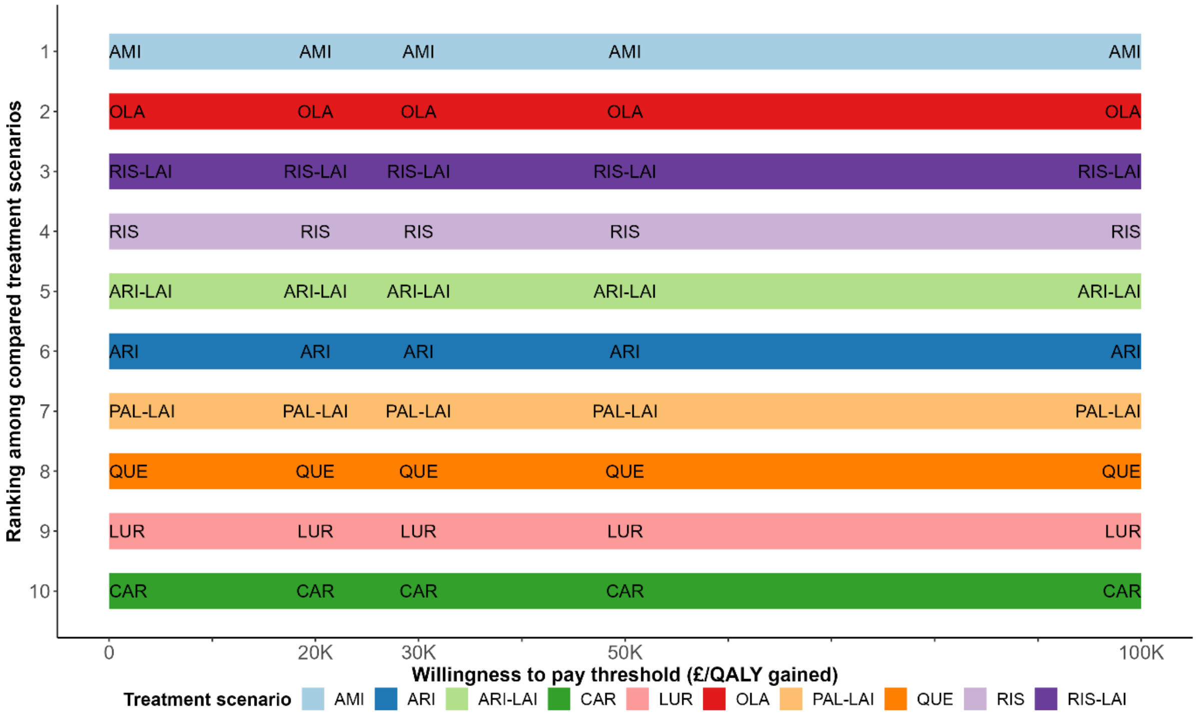

Treatment is an oral one if its name is not followed by “LAI”; AMI, amisulpride; ARI, aripiprazole; CAR, cariprazine; LAI, long-acting injection; LUR, lurasidone; OLA, olanzapine; PAL, paliperidone; QALY, quality-adjusted life year; QUE, quetiapine; RIS, risperidone;

Supplementary figure 7. Scenario analyses results including olanzapine-LAI into the comparison among first-line antipsychotics in first-episode schizophrenia

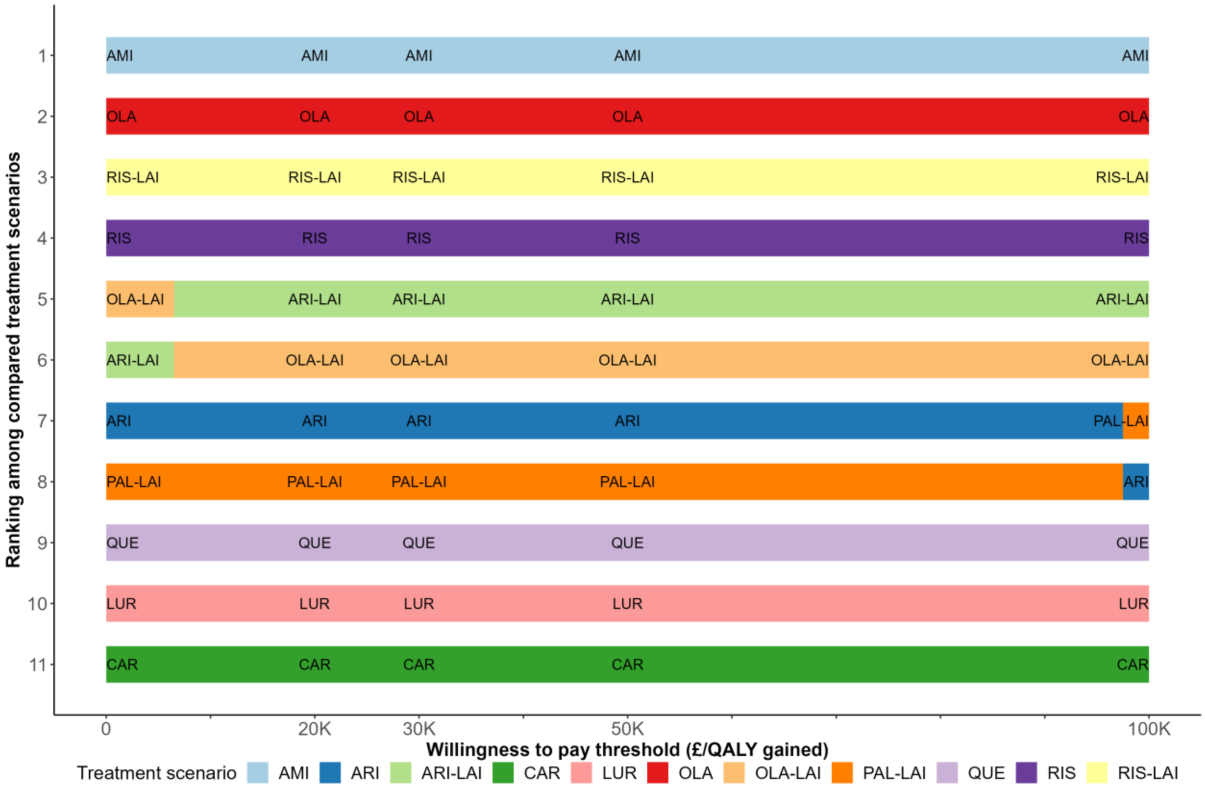

We used different inputs of treatment profiles for olanzapine-LAI than oral olanzapine in risk ratio on relapse prevention (0.29 vs. 0.20), annual probability of discontinuation (0.35 vs. 0.27), and annual drug costs (£3426 vs. £23) based on the recent NMA[93] and BNF drug tariff[120].

Treatment is an oral one if its name is not followed by "LAI"; AMI, amisulpride; ARI, aripiprazole; CAR, cariprazine; LAI, long-acting injection; LUR, lurasidone; OLA, olanzapine; PAL, paliperidone; QALY, quality-adjusted life year; QUE, quetiapine; RIS, risperidone;

Supplementary references

1. Smith J, Griffiths LA, Band M, Horne D. Cardiometabolic Risk in First Episode Psychosis Patients. *Front Endocrinol (Lausanne)* 2020; **11**: 564240.
2. Barnett AH, Millar HL, Loze JY, L'Italien GJ, van Baardewijk M, Knapp M. UK cost-consequence analysis of aripiprazole in schizophrenia: diabetes and coronary heart disease risk projections (STAR study). *Eur Arch Psychiatry Clin Neurosci* 2009; **259**(4): 239-47.
3. Osborn DP, Hardoon S, Omar RZ, et al. Cardiovascular risk prediction models for people with severe mental illness: results from the prediction and management of cardiovascular risk in people with severe mental illnesses (PRIMROSE) research program. *JAMA Psychiatry* 2015; **72**(2): 143-51.
4. Gilmer TP, Dolder CR, Lacro JP, et al. Adherence to treatment with antipsychotic medication and health care costs among Medicaid beneficiaries with schizophrenia. *Am J Psychiatry* 2004; **161**(4): 692-9.
5. Smith DJ, Langan J, McLean G, Guthrie B, Mercer SW. Schizophrenia is associated with excess multiple physical-health comorbidities but low levels of recorded cardiovascular disease in primary care: cross-sectional study. *BMJ Open* 2013; **3**(4).
6. Zhao J, Jiang K, Li Q, et al. Cost-effectiveness of olanzapine in the first-line treatment of schizophrenia in China. *J Med Econ* 2019; **22**(5): 439-46.
7. Nemeth B, Bendes R, Nagy B, et al. Cost-utility analysis of cariprazine compared to risperidone among patients with negative symptoms of schizophrenia. *Health Policy and Technology* 2019; **8**(1): 84-91.
8. El Khoury AC, Pilon D, Morrison L, et al. The prospective economic impact of once monthly paliperidone palmitate versus oral atypical antipsychotics in Medicaid patients with schizophrenia. *Curr Med Res Opin* 2019; **35**(3): 395-405.
9. Thavornwattanayong W, Lertsirimunkong J, Thongkerd N, Pitakthanin N, Wettayanon P, Pongjakpanit H. Cost-effectiveness analysis of aripiprazole compared with risperidone in the treatment of acute schizophrenia patients in Thailand. *Thai Journal of Pharmaceutical Sciences* 2018; **42**(3): 169-75.
10. Nuhoho S, Saad A, Saumell G, Ribes D, El Khoury AC. Economic evaluation of paliperidone palmitate once monthly for treating chronic schizophrenia patients in the United Arab Emirates. *Current Medical Research & Opinion* 2018; **34**(4): 601-11.
11. Aigbogun MS, Liu S, Kamat SA, Sapin C, Duhig AM, Citrome L. Relapse prevention: a cost-effectiveness analysis of brexpiprazole treatment in adult patients with schizophrenia in the USA. *ClinicoEcon* 2018; **10**: 443-56.
12. Einarson TR, Bereza BG, Tedouri F, Van Impe K, Denee TR, Dries PJT. Cost-effectiveness of 3-month paliperidone therapy for chronic schizophrenia in the Netherlands. *Journal of Medical Economics* 2017; **20**(11): 1187-99.
13. Einarson TR, Bereza BG, Garcia Llinares I, Gonzalez Martin Moro B, Tedouri F, Van Impe K. Cost-effectiveness of 3-month paliperidone treatment for chronic schizophrenia in Spain. *Journal of Medical Economics* 2017; **20**(10): 1039-47.
14. Rajagopalan K, Trueman D, Crowe L, Squirrell D, Loebel A. Cost-Utility Analysis of Lurasidone Versus Aripiprazole in Adults with Schizophrenia. *Pharmacoeconomics* 2016; **34**(7): 709-21.
15. Lin L, Zhao YJ, Zhou HJ, et al. Comparative cost-effectiveness of 11 oral antipsychotics for relapse prevention in schizophrenia within Singapore using effectiveness estimates from a network meta-analysis. *Int Clin Psychopharmacol* 2016; **31**(2): 84-92.
16. Einarson TR, Pudas H, Goswami P, van Impe K, Bereza BG. Pharmacoeconomics of long-acting atypical antipsychotics for acutely relapsed chronic schizophrenia in Finland. *J Med Econ* 2016; **19**(2): 111-20.
17. Druais S, Doutriaux A, Cognet M, et al. Cost Effectiveness of Paliperidone Long-Acting Injectable Versus Other Antipsychotics for the Maintenance Treatment of Schizophrenia in France. *Pharmacoeconomics* 2016; **34**(4): 363-91.

18. Lubinga SJ, Mutamba BB, Nganizi A, Babigumira JB. A Cost-effectiveness Analysis of Antipsychotics for Treatment of Schizophrenia in Uganda. *Appl Health Econ Health Policy* 2015; **13**(5): 493-506.
19. Anh NQ, Linh BN, Ha NT, Phanthunane P, Huong NT. Schizophrenia interventions in Vietnam: primary results from a cost-effectiveness study. *Glob Public Health* 2015; **10 Supppl 1**: S21-39.
20. Park T, Kuntz KM. Cost-effectiveness of second-generation antipsychotics for the treatment of schizophrenia. *Value Health* 2014; **17**(4): 310-9.
21. National Institute for Health and Clinical Excellence. Psychosis and schizophrenia in adults: prevention and management. *NICE Guideline CG178;* 2014; **Available from: https://www.nice.org.uk/guidance/cg178. Last accessed 04 April 2018.**
22. Lachaine J, Beauchemin C, Mathurin K, Gilbert D, Beillat M. Cost-effectiveness of asenapine in the treatment of schizophrenia in Canada. *J Med Econ* 2014; **17**(4): 296-304.
23. Frey S, Linder R, Juckel G, Stargardt T. Cost-effectiveness of long-acting injectable risperidone versus flupentixol decanoate in the treatment of schizophrenia: a Markov model parameterized using administrative data. *Eur J Health Econ* 2014; **15**(2): 133-42.
24. Einarson TR, Vicente C, Zilbershtein R, et al. Pharmacoeconomics of depot antipsychotics for treating chronic schizophrenia in Sweden. *Nord J Psychiatry* 2014; **68**(6): 416-27.
25. Dilla T, Moller J, O'Donohoe P, et al. Long-acting olanzapine versus long-acting risperidone for schizophrenia in Spain - a cost-effectiveness comparison. *BMC Psychiatry* 2014; **14**: 298.
26. Citrome L, Kamat SA, Sapin C, et al. Cost-effectiveness of aripiprazole once-monthly compared with paliperidone palmitate once-monthly injectable for the treatment of schizophrenia in the United States. *J Med Econ* 2014; **17**(8): 567-76.
27. Zeidler J, Mahlich J, Greiner W, Heres S. Cost effectiveness of paliperidone palmitate for the treatment of schizophrenia in Germany. *Appl Health Econ Health Policy* 2013; **11**(5): 509-21.
28. Rajagopalan K, Hassan M, O'Day K, Meyer K, Grossman F. Cost-effectiveness of lurasidone vs aripiprazole among patients with schizophrenia who have previously failed on an atypical antipsychotic: an indirect comparison of outcomes from clinical trial data. *J Med Econ* 2013; **16**(7): 951-61.
29. O'Day K, Rajagopalan K, Meyer K, Pikalov A, Loebel A. Long-term cost-effectiveness of atypical antipsychotics in the treatment of adults with schizophrenia in the US. *Clinicoecon Outcomes Res* 2013; **5**: 459-70.
30. Jukic V, Jakovljevic M, Filipcic I, et al. Cost-Utility Analysis of Depot Atypical Antipsychotics for Chronic Schizophrenia in Croatia. *Value in Health Regional Issues* 2013; **2**(2): 181-8.
31. Einarson TR, Zilbershtein R, Skoupa J, Vesela S, Garg M, Hemels ME. Economic and clinical comparison of atypical depot antipsychotic drugs for treatment of chronic schizophrenia in the Czech Republic. *J Med Econ* 2013; **16**(9): 1089-95.
32. Einarson TR, Vicente C, Zilbershtein R, et al. Pharmacoeconomic analysis of paliperidone palmitate versus olanzapine pamoate for chronic schizophrenia in Norway. *Acta Neuropsychiatr* 2013; **25**(2): 85-94.
33. Einarson TR, Pudas H, Zilbershtein R, et al. Cost-effectiveness analysis of atypical long-acting antipsychotics for treating chronic schizophrenia in Finland. *J Med Econ* 2013; **16**(9): 1096-105.
34. Treur M, Baca E, Bobes J, et al. The cost-effectiveness of paliperidone extended release in Spain. *J Med Econ* 2012; **15 Suppl 1**: 26-34.
35. Mehnert A, Nicholl D, Pudas H, Martin M, McGuire A. Cost effectiveness of paliperidone palmitate versus risperidone long-acting injectable and olanzapine pamoate for the treatment of patients with schizophrenia in Sweden. *J Med Econ* 2012; **15**(5): 844-61.
36. Kim B-R-M, Lee T-J, Lee H-J, Park B-H, Yang B-M. Cost-Effectiveness of Sertindole among Atypical Antipsychotics in the Treatment of Schizophrenia in South Korea. *Value in Health Regional Issues* 2012; **1**(1): 59-65.
37. Graham CN, Mauskopf JA, Lawson AH, Ascher-Svanum H, Bruhn D. Updating and confirming an industry-sponsored pharmacoeconomic model: comparing two antipsychotics in the treatment of schizophrenia. *Value Health* 2012; **15**(1): 55-64.

38. Garcia-Ruiz AJ, Perez-Costillas L, Montesinos AC, Alcalde J, Oyaguez I, Casado MA. Cost-effectiveness analysis of antipsychotics in reducing schizophrenia relapses. *Health Econ Rev* 2012; **2**(1): 8.
39. Einarson TR, Geitona M, Chaidemenos A, et al. Pharmacoeconomic analysis of paliperidone palmitate for treating schizophrenia in Greece. *Ann Gen Psychiatry* 2012; **11**(1): 18.
40. Edwards NC, Muser E, Doshi D, Fastenau J. The threshold rate of oral atypical anti-psychotic adherence at which paliperidone palmitate is cost saving. *J Med Econ* 2012; **15**(4): 623-34.
41. Ascher-Svanum H, Furiak NM, Lawson AH, et al. Cost-effectiveness of several atypical antipsychotics in orally disintegrating tablets compared with standard oral tablets in the treatment of schizophrenia in the United States. *J Med Econ* 2012; **15**(3): 531-47.
42. Phanthunane P, Vos T, Whiteford H, Bertram M. Cost-effectiveness of pharmacological and psychosocial interventions for schizophrenia. *Cost Eff Resour Alloc* 2011; **9**: 6.
43. National Institute for Health and Clinical Excellence. Aripiprazole for schizophrenia in people aged 15 to 17 years. *NICE Technology apprasial guidance TA213;* 2011; **Available from: www.nice.org.uk/guidance/TA213. Last accessed 04 April 2018**.
44. Lindstrom E, Eberhard J, Fors BM, Hansen K, Sapin C. A pharmacoeconomic analysis of sertindole in the treatment of schizophrenia in Sweden. *Nord J Psychiatry* 2011; **65**(6): 403-13.
45. Kasteng F, Eriksson J, Sennfalt K, Lindgren P. Metabolic effects and cost-effectiveness of aripiprazole versus olanzapine in schizophrenia and bipolar disorder. *Acta Psychiatr Scand* 2011; **124**(3): 214-25.
46. Furiak NM, Ascher-Svanum H, Klein RW, et al. Cost-effectiveness of olanzapine long-acting injection in the treatment of patients with schizophrenia in the United States: a micro-simulation economic decision model. *Curr Med Res Opin* 2011; **27**(4): 713-30.
47. McIntyre RS, Cragin L, Sorensen S, Naci H, Baker T, Roussy JP. Comparison of the metabolic and economic consequences of long-term treatment of schizophrenia using ziprasidone, olanzapine, quetiapine and risperidone in Canada: a cost-effectiveness analysis. *J Eval Clin Pract* 2010; **16**(4): 744-55.
48. Hensen M, Heeg B, Lothgren M, van Hout B. Cost effectiveness of long-acting risperidone in Sweden. *Appl Health Econ Health Policy* 2010; **8**(5): 327-41.
49. Yang L, Li M, Tao LB, Zhang M, Nicholl MD, Dong P. Cost-effectiveness of long-acting risperidone injection versus alternative atypical antipsychotic agents in patients with schizophrenia in China. *Value Health* 2009; **12 Suppl 3**: S66-9.
50. Treur M, Heeg B, Moller HJ, Schmeding A, van Hout B. A pharmaco-economic analysis of patients with schizophrenia switching to generic risperidone involving a possible compliance loss. *BMC Health Serv Res* 2009; **9**: 32.
51. Lindner LM, Marasciulo AC, Farias MR, Grohs GE. Economic evaluation of antipsychotic drugs for schizophrenia treatment within the Brazilian Healthcare System. *Rev Saude Publica* 2009; **43 Suppl 1**: 62-9.
52. Furiak NM, Ascher-Svanum H, Klein RW, et al. Cost-effectiveness model comparing olanzapine and other oral atypical antipsychotics in the treatment of schizophrenia in the United States. *Cost Eff Resour Alloc* 2009; **7**: 4.
53. Heeg BM, Antunes J, Figueira ML, et al. Cost-effectiveness and budget impact of long-acting risperidone in Portugal: a modeling exercise. *Curr Med Res Opin* 2008; **24**(2): 349-58.
54. Heeg B, Buskens E, Botteman M, et al. The cost-effectiveness of atypicals in the UK. *Value Health* 2008; **11**(7): 1007-21.
55. Geitona M, Kousoulakou H, Ollandezos M, Athanasakis K, Papanicolaou S, Kyriopoulos I. Costs and effects of paliperidone extended release compared with alternative oral antipsychotic agents in patients with schizophrenia in Greece: a cost effectiveness study. *Ann Gen Psychiatry* 2008; **7**: 16.
56. Edwards NC, Pesa J, Meletiche DM, et al. One-year clinical and economic consequences of oral atypical antipsychotics in the treatment of schizophrenia. *Curr Med Res Opin* 2008; **24**(12): 3341-55.
57. Davies A, Vardeva K, Loze JY, L'Italien G J, Sennfalt K, Baardewijk M. Cost-effectiveness of atypical antipsychotics for the management of schizophrenia in the UK. *Curr Med Res Opin* 2008; **24**(11): 3275-85.

58. Damen J, Thuresson PO, Heeg B, Lothgren M. A pharmacoeconomic analysis of compliance gains on antipsychotic medications. *Appl Health Econ Health Policy* 2008; **6**(4): 189-97.
59. Colombo GL, Caruggi M, Di Matteo S, Rossi A. An economic evaluation of aripiprazole vs olanzapine adapted to the Italian setting using outcomes of metabolic syndrome and risk for diabetes in patients with schizophrenia. *Neuropsychiatr Dis Treat* 2008; **4**(5): 967-76.
60. Alexeyeva I, Mauskopf J, Earnshaw SR, et al. Comparing olanzapine and ziprasidone in the treatment of schizophrenia: a case study in modeling. *Journal of Medical Economics* 2008; **4**(1-4): 179-92.
61. Obradovic M, Mrhar A, Kos M. Cost-effectiveness of antipsychotics for outpatients with chronic schizophrenia. *Int J Clin Pract* 2007; **61**(12): 1979-88.
62. Bounthavong M, Okamoto MP. Decision analysis model evaluating the cost-effectiveness of risperidone, olanzapine and haloperidol in the treatment of schizophrenia. *J Eval Clin Pract* 2007; **13**(3): 453-60.
63. Bernardo M, Azanza JR, Rubio-Terres C, Rejas J. Cost-effectiveness analysis of the prevention of relapse of schizophrenia in the longitudinal study Ziprasidone Extended Use in Schizophrenia (ZEUS). *Actas Esp Psiquiatr* 2007; **35**(4): 259-62.
64. Beard SM, Maciver F, Clouth J, Ruther E. A decision model to compare health care costs of olanzapine and risperidone treatment for schizophrenia in Germany. *Eur J Health Econ* 2006; **7**(3): 165-72.
65. Yang YK, Tarn YH, Wang TY, et al. Pharmacoeconomic evaluation of schizophrenia in Taiwan: model comparison of long-acting risperidone versus olanzapine versus depot haloperidol based on estimated costs. *Psychiatry Clin Neurosci* 2005; **59**(4): 385-94.
66. Perlis RH, Ganz DA, Avorn J, et al. Pharmacogenetic testing in the clinical management of schizophrenia: a decision-analytic model. *J Clin Psychopharmacol* 2005; **25**(5): 427-34.
67. Magnus A, Carr V, Mihalopoulos C, Carter R, Vos T. Assessing cost-effectiveness of drug interventions for schizophrenia. *Aust N Z J Psychiatry* 2005; **39**(1-2): 44-54.
68. Laux G, Heeg B, van Hout BA, Mehnert A. Costs and effects of long-acting risperidone compared with oral atypical and conventional depot formulations in Germany. *Pharmacoeconomics* 2005; **23 Suppl 1**: 49-61.
69. Kongsakon R, Leelahanaj T, Price N, Birinyi-Strachan L, Davey P. Cost analysis of the treatment of schizophrenia in Thailand: a simulation model comparing olanzapine, risperidone, quetiapine, ziprasidone and haloperidol. *J Med Assoc Thai* 2005; **88**(9): 1267-77.
70. Heeg B, Buskens E, Knapp M, et al. Modelling the treated course of schizophrenia: development of a discrete event simulation model. *Pharmacoeconomics* 2005; **23 Suppl 1**: 17-33.
71. Greenhalgh J, Knight C, Hind D, Beverley C, Walters S. Clinical and cost-effectiveness of electroconvulsive therapy for depressive illness, schizophrenia, catatonia and mania: systematic reviews and economic modelling studies. *Health Technol Assess* 2005; **9**(9): 1-156, iii-iv.
72. Edwards NC, Locklear JC, Rupnow MF, Diamond RJ. Cost effectiveness of long-acting risperidone injection versus alternative antipsychotic agents in patients with schizophrenia in the USA. *Pharmacoeconomics* 2005; **23 Suppl 1**: 75-89.
73. De Graeve D, Smet A, Mehnert A, et al. Long-acting risperidone compared with oral olanzapine and haloperidol depot in schizophrenia: a Belgian cost-effectiveness analysis. *Pharmacoeconomics* 2005; **23 Suppl 1**: 35-47.
74. Chue PS, Heeg B, Buskens E, van Hout BA. Modelling the impact of compliance on the costs and effects of long-acting risperidone in Canada. *Pharmacoeconomics* 2005; **23 Suppl 1**: 62-74.
75. Wang PS, Ganz DA, Benner JS, Glynn RJ, Avorn J. Should clozapine continue to be restricted to third-line status for schizophrenia?: a decision-analytic model. *J Ment Health Policy Econ* 2004; **7**(2): 77-85.
76. Vera-Llonch M, Delea TE, Richardson E, Rupnow M, Grogg A, Oster G. Outcomes and costs of risperidone versus olanzapine in patients with chronic schizophrenia or schizoaffective disorders: a Markov model. *Value Health* 2004; **7**(5): 569-84.
77. Layton S, Barbeau M. Generic replacement of clozapine: a simple decision model from a Canadian perspective. *Curr Med Res Opin* 2004; **20**(4): 453-9.

78. Emsley R, Booysen F. Cost-effectiveness of an atypical conventional antipsychotic in South Africa: An economic evaluation of quetiapine versus haloperidol in the treatment of patients partially responsive to previous antipsychotics. *South African Journal of Psychiatry* 2004; **10**(3): 7.
79. Bobes J, Canas F, Rejas J, Mackell J. Economic consequences of the adverse reactions related with antipsychotics: an economic model comparing tolerability of ziprasidone, olanzapine, risperidone, and haloperidol in Spain. *Prog Neuropsychopharmacol Biol Psychiatry* 2004; **28**(8): 1287-97.
80. Mortimer A, Williams P, Meddis D. Impact of side-effects of atypical antipsychotics on non-compliance, relapse and cost. *J Int Med Res* 2003; **31**(3): 188-96.
81. Ganguly R, Miller LS, Martin BC. Future employability, a new approach to cost-effectiveness analysis of antipsychotic therapy. *Schizophr Res* 2003; **63**(1-2): 111-9.
82. Bagnall AM, Jones L, Ginnelly L, et al. A systematic review of atypical antipsychotic drugs in schizophrenia. *Health Technol Assess* 2003; **7**(13): 1-193.
83. Tilden D, Aristides M, Meddis D, Burns T. An economic assessment of quetiapine and haloperidol in patients with schizophrenia only partially responsive to conventional antipsychotics. *Clin Ther* 2002; **24**(10): 1648-67.
84. Palmer CS, Brunner E, Ruiz-Flores LG, Paez-Agraz F, Revicki DA. A cost-effectiveness clinical decision analysis model for treatment of Schizophrenia. *Arch Med Res* 2002; **33**(6): 572-80.
85. Hansen K, Francois C, Toumi M, Lancon C. A pharmacoeconomic evaluation of zuclopenthixol compared with haloperidol and risperidone in the treatment of schizophrenia. *Eur J Health Econ* 2002; **3**(3): 173-9.
86. Oh PI, Lanctôt KL, Mittmann N, Iskedjian M, Einarson TR. Cost-utility of risperidone compared with standard conventional antipsychotics in chronic schizophrenia. *Journal of Medical Economics* 2001; **4**(1-4): 137-56.
87. Oh PI, Iskedjian M, Addis A, Lanctot K, Einarson TR. Pharmacoeconomic evaluation of clozapine in treatment-resistant schizophrenia: a cost-utility analysis. *Can J Clin Pharmacol* 2001; **8**(4): 199-206.
88. Lecomte P, De Hert M, van Dijk M, Nuijten M, Nuyts G, Persson U. A 1-year cost-effectiveness model for the treatment of chronic schizophrenia with acute exacerbations in Belgium. *Value Health* 2000; **3**(1): 1-11.
89. Almond S, O'Donnell O. Cost analysis of the treatment of schizophrenia in the UK. A simulation model comparing olanzapine, risperidone and haloperidol. *Pharmacoeconomics* 2000; **17**(4): 383-9.
90. Zhou J, Millier A, Toumi M. Systematic review of pharmacoeconomic models for schizophrenia. *J Mark Access Health Policy* 2018; **6**(1): 1508272.
91. Zhou J. Development of a discrete event simulation model for health economic evaluation of interventions for patients with schizophrenia. 2020. Thesis availvable from https://theses.fr/2020AIXM0090 (Accessed at 2024/07/25).
92. National Health Service Business Services Authority. Prescription cost analysis - England 2020/2021. 2021. https://www.nhsbsa.nhs.uk/statistical-collections/prescription-cost-analysis-england/prescription-cost-analysis-england-202021 (accessed 2023/04/01.
93. Schneider-Thoma J, Chalkou K, Dorries C, et al. Comparative efficacy and tolerability of 32 oral and long-acting injectable antipsychotics for the maintenance treatment of adults with schizophrenia: a systematic review and network meta-analysis. *Lancet* 2022; **399**(10327): 824-36.
94. Ostuzzi G, Bertolini F, Tedeschi F, et al. Oral and long-acting antipsychotics for relapse prevention in schizophrenia-spectrum disorders: a network meta-analysis of 92 randomized trials including 22,645 participants. *World Psychiatry* 2022; **21**(2): 295-307.
95. Essali A, Al-Haj Haasan N, Li C, Rathbone J. Clozapine versus typical neuroleptic medication for schizophrenia. *Cochrane Database Syst Rev* 2009; **2009**(1): CD000059.
96. National Institute for Health and Care Excellence. Psychosis and schizophrenia in adults: prevention and management (CG178). Available from: https://www.nice.org.uk/guidance/cg178. Accessed on 2023.01.01, 2014.
97. Kishimoto T, Hagi K, Nitta M, Kane JM, Correll CU. Long-term effectiveness of oral second-generation antipsychotics in patients with schizophrenia and related disorders: a systematic review and meta-analysis of direct head-to-head comparisons. *World Psychiatry* 2019; **18**(2): 208-24.

98. Leucht S, Cipriani A, Spineli L, et al. Comparative efficacy and tolerability of 15 antipsychotic drugs in schizophrenia: a multiple-treatments meta-analysis. *Lancet* 2013; **382**(9896): 951-62.
99. Schmidt-Kraepelin C, Feyerabend S, Engelke C, et al. Amisulpride and olanzapine combination treatment versus each monotherapy in acutely ill patients with schizophrenia in Germany (COMBINE): a double-blind randomised controlled trial. *Lancet Psychiatry* 2022; **9**(4): 291-306.
100. Serretti A, Chiesa A. A meta-analysis of sexual dysfunction in psychiatric patients taking antipsychotics. *Int Clin Psychopharmacol* 2011; **26**(3): 130-40.
101. Clayton AH, Tsai J, Mao Y, Pikalov A, Loebel A. Effect of Lurasidone on Sexual Function in Major Depressive Disorder Patients With Subthreshold Hypomanic Symptoms (Mixed Features): Results From a Placebo-Controlled Trial. *J Clin Psychiatry* 2018; **79**(5).
102. Potkin SG, Loze JY, Forray C, et al. Reduced sexual dysfunction with aripiprazole once-monthly versus paliperidone palmitate: results from QUALIFY. *Int Clin Psychopharmacol* 2017; **32**(3): 147-54.
103. Carbon M, Kane JM, Leucht S, Correll CU. Tardive dyskinesia risk with first- and second-generation antipsychotics in comparative randomized controlled trials: a meta-analysis. *World Psychiatry* 2018; **17**(3): 330-40.
104. Munro J, O'Sullivan D, Andrews C, Arana A, Mortimer A, Kerwin R. Active monitoring of 12,760 clozapine recipients in the UK and Ireland. Beyond pharmacovigilance. *Br J Psychiatry* 1999; **175**: 576-80.
105. Deepak TS, Raveesh BN, Parashivamurthy BM, Kumar MN, Majgi SM, Nagesh HN. Clinical Assessment of Weight Gain with Atypical Antipsychotics - Blonanserin vs Amisulpride. *J Clin Diagn Res* 2015; **9**(6): FC07-10.
106. Pillinger T, McCutcheon RA, Vano L, et al. Comparative effects of 18 antipsychotics on metabolic function in patients with schizophrenia, predictors of metabolic dysregulation, and association with psychopathology: a systematic review and network meta-analysis. *Lancet Psychiatry* 2020; **7**(1): 64-77.
107. Greger J, Aladeen T, Lewandowski E, et al. Comparison of the Metabolic Characteristics of Newer Second Generation Antipsychotics: Brexpiprazole, Lurasidone, Asenapine, Cariprazine, and Iloperidone With Olanzapine as a Comparator. *J Clin Psychopharmacol* 2021; **41**(1): 5-12.
108. Gupta A, Dadheech G, Yadav D, Sharma P, Gautam S. Metabolic issues in schizophrenic patients receiving antipsychotic treatment. *Indian J Clin Biochem* 2014; **29**(2): 196-201.
109. Wilson PW, Meigs JB, Sullivan L, Fox CS, Nathan DM, D'Agostino RB, Sr. Prediction of incident diabetes mellitus in middle-aged adults: the Framingham Offspring Study. *Arch Intern Med* 2007; **167**(10): 1068-74.
110. D'Agostino RB, Russell MW, Huse DM, et al. Primary and subsequent coronary risk appraisal: new results from the Framingham study. *Am Heart J* 2000; **139**(2 Pt 1): 272-81.
111. D'Agostino RB, Wolf PA, Belanger AJ, Kannel WB. Stroke risk profile: adjustment for antihypertensive medication. The Framingham Study. *Stroke* 1994; **25**(1): 40-3.
112. Reininghaus U, Dutta R, Dazzan P, et al. Mortality in schizophrenia and other psychoses: a 10-year follow-up of the ÆSOP first-episode cohort. *Schizophr Bull* 2015; **41**(3): 664-73.
113. Seminog OO, Scarborough P, Wright FL, Rayner M, Goldacre MJ. Determinants of the decline in mortality from acute stroke in England: linked national database study of 795 869 adults. *BMJ* 2019; **365**: l1778.
114. Asaria P, Elliott P, Douglass M, et al. Acute myocardial infarction hospital admissions and deaths in England: a national follow-back and follow-forward record-linkage study. *Lancet Public Health* 2017; **2**(4): e191-e201.
115. Lenert LA, Sturley AP, Rapaport MH, Chavez S, Mohr PE, Rupnow M. Public preferences for health states with schizophrenia and a mapping function to estimate utilities from positive and negative symptom scale scores. *Schizophr Res* 2004; **71**(1): 155-65.
116. Weeks JC, Tierney MR, Weinstein MC. Cost effectiveness of prophylactic intravenous immune globulin in chronic lymphocytic leukemia. *N Engl J Med* 1991; **325**(2): 81-6.
117. Millier A, Amri I, Boyer L, Auquier P, Toumi M. Utility decrements associated with side effects in schizophrenia. *J Med Econ* 2014; **17**(12): 853-61.

118. Briggs A, Wild D, Lees M, et al. Impact of schizophrenia and schizophrenia treatment-related adverse events on quality of life: direct utility elicitation. *Health Qual Life Outcomes* 2008; **6**: 105.
119. Clarke P, Gray A, Holman R. Estimating utility values for health states of type 2 diabetic patients using the EQ-5D (UKPDS 62). *Med Decis Making* 2002; **22**(4): 340-9.
120. Joint Formulary Committee. British National Formulary. 76th Ed., London: BMJ Group and Pharmaceutical Press.; 2020.
121. National Health Service England. National Schedule of NHS Costs 2019/20. 2021. https://www.england.nhs.uk/publication/2019-20-national-cost-collection-data-publication/ (accessed 2023/09/30.
122. Personal Social Services Research Unit (PSSRU). Unit Costs of Health and Social Care 2020 - Community-based Health Care Staff. 2020. https://www.pssru.ac.uk/pub/uc/uc2020/2-communityhcstaff.pdf (accessed 2021/09/30).
123. Zhou J, Wu R, Williams C, et al. Prediction Models for Individual-Level Healthcare Costs Associated with Cardiovascular Events in the UK. *Pharmacoeconomics* 2023.
